# The self-medication behaviors of residents and the factors related to the consideration of drug efficacy and safety—A cross-sectional study in China

**DOI:** 10.3389/fphar.2023.1072917

**Published:** 2023-02-28

**Authors:** Pu Ge, Zi-Wei Zhang, Jin-Zi Zhang, Ke Lyu, Yu-Yao Niu, Yu-Ting Tong, Ping Xiong, Rong Ling, Qi-Yu Li, Wen-Li Yu, He-Wei Min, Yu-Qian Deng, Yu-Jia Wang, Xiao-Nan Sun, Xin-Ying Sun, Lian Yu, Yi-Bo Wu

**Affiliations:** ^1^ Institute of Chinese Medical Sciences, University of Macau, Macao, China; ^2^ School of Public Health, Peking University, Beijing, China; ^3^ School of Humanities and Social Sciences, Harbin Medical University, Harbin, China; ^4^ China Medical University, Liaoning, China; ^5^ Department of English, Faculty of Arts and Humanities, University of Macau, Macao, China; ^6^ School of Public Health, Shandong University, Jinan, China; ^7^ Jilin University School of Pharmaceutical Sciences, Jilin University, Changchun, China; ^8^ School of Humanities and management, Jinzhou Medical University, Jinzhou, China; ^9^ School of Foreign Languages, Weifang University of Science and Technology, Weifang, China; ^10^ Xiangya School of Nursing, Central South University, Changsha, China; ^11^ Health Care system Reform and Development Institute, School of Public Health, Xi’an Jiaotong University Health Science Center, Xi’an, China

**Keywords:** self-medication, drug efficacy, drug safety, over-the-counter drugs, China, cross-sectional study

## Abstract

**Background:** Over-the-counter (OTC) drugs facilitates residents self-medication. However, inappropriate self-medications have become a serious problem in China and even all over the world.

**Objectives:** To make an investigation on the current status of Chinese residents’ self-medication behaviors and important considerations, and to explore the factors related to the considerations of drug efficacy and safety.

**Design:** A quantitative, cross-sectional study.

**Methods:** Multi-stage sampling was used to conduct a cross-sectional investigation in China 22 provinces, 5 autonomous regions and 4 municipalities directly under the Central Government. State that an interviewer-administrated questionnaire, was used for data collection. The questionnaire that was used in the investigation included demographic sociological characteristics, health literacy scale-short form (HLS-SF), the 10-item Big Five Inventory (BFI-10), the EuroQol-5D visual analogue scale (EQ-5D VAS), self-medication status and important considerations when self-medicating. Descriptive statistics were performed, and the Chi-square test was used for univariate analysis. Log-binomial regression was used for multivariate analysis on whether residents regard drug efficacy or safety as an important consideration.

**Results:** 9256 respondents were included in the data analysis. The self-medication rate of Chinese adults was as high as 99.1%. Paracetamol and other analgesics were the most common types of OTC medication that respondents purchased, followed by vitamins/minerals. Medical staff recommendations, drug safety and efficacy were the top three important considerations. The residents in the east, central and western regions who consider safety is 63.5%, 61.5%, and 66.8% respectively. The proportion of curative effect was 60.2%, 55.7%, and 61.4% respectively. Log-binomial regression showed that western respondents, retired people, those who mainly used ways including basic medical insurance for employees, commercial medical insurance, free medical treatment to cover their medical cost, respondents with high neuroticism, high health literacy were more likely to consider drug safety as an important factor (*p* < 0.05). Eastern respondents, employed, main way of medical expenses borne was Out-of-pocket Payment, those with chronic disease were more likely to consider drug efficacy as an important factor (*p* < 0.05). Female, respondents with high levels of agreeableness, conscientiousness, openness, and self-rated health status were more likely to regard both drug safety and efficacy as important considerations (*p* < 0.05).

**Conclusion:** Self-medication is practiced by most Chinese adults. Whether Chinese adults take drug efficacy or safety as an important consideration is related to their demographic and sociological characteristics, Big Five personality characteristics, health literacy and self-assessed health status. There is a need to strengthen the management of OTC drugs and public education about self-medication.

## Highlights

What does this paper contribute to the wider global clinical community?

1. Providing a reference for decision-makers to formulate policies.
2. Actively strengthening the management of OTC drugs and public health education on self-medication.
3. The health department needs to establish a system that promotes the partnership between physicians, patients and pharmacists.
4. Mobilizing relevant media to publicize drug-related knowledge and common sense through various forms of media.
5. Encouraging medical staff to provide more guidance on the safety and efficacy of drugs to help patients choose drugs.

## 1 Introduction

The World Health Organization defined self-medication as the use of drugs to treat self-diagnosed disorders or symptoms, or the intermittent or continuous use of prescribed medication to treat a chronic or recurrent disease or symptoms (World Health Organization, 2000). A large portion of the public uses self-medication as an important way to treat minor illnesses or relieve symptoms A study in Iran found that the self-medication rate was as high as 90% (Fereidouni et al., 2019). In a study in the United States, the majority of the population (87.0%) took at least one OTC drug (Stoehr et al., 1997). Self-medication has become more prevalent as the COVID-19 outbreak has led to fewer people going out and going to the hospital to avoid infection.

The most widely self-medicated substances are OTC drugs, which are used to treat common health issues at home. These drugs do not require a doctor’s prescription and are available in some countries at supermarkets and convenience stores (WORLD SELF-MEDICATION INDUSTRY, 2022). The U.S. Food and Drug Administration (FDA) defines OTC drugs as those that can be purchased without a medical prescription (U.S. Food and drug administration, 2022). In China, there are also categories governing prescription and OTC drugs (Lei et al., 2018).

The growing variety of OTC drugs facilitates residents’ self-medication behavior. The appropriateness of residents’ self-medication behavior has a significant impact on the efficacy and safety of the drugs. Appropriate use of OTC drugs can timely relieve minor symptoms of chronic diseases, such as upper respiratory tract infection, headache, atopic dermatitis and stomach pain, etc., thereby reducing the economic and time cost of patient care and reducing the pressure on medical institutions and the burden on medical insurance (Lei et al., 2018). However, inappropriate self-medication behaviors can delay or obscure the diagnosis of patients’ serious diseases and increase the risk of potential adverse effects in patients. Inappropriate self-medication has caused harm to some residents and is increasingly becoming a serious public problem globally (Tesfamariam et al., 2019). In China, about 2.5 million people are hospitalized each year due to inappropriate self-medication, and about 100,000 people die from adverse drug reactions (Zhao et al., 2017).

Residents purchase OTC drugs based on a variety of factors, such as drug price, efficacy, and safety, as well as advice from medical staff, family, friends, and so on (Shrestha et al., 2022; Tavares et al., 2022). Personality represents a series of personality characteristics, thinking patterns and habitual behaviors within an individual, and influences the individual’s response to external stimuli and interaction with others in society (Getzmann et al., 2021). Health literacy is an individual’s ability to acquire health information and understand disease-related knowledge. Good health literacy is conducive to residents’ accurate judgment and rational use of health information, so as to maintain their own health (DE Wit et al., 2017). It has been found that personality characteristics and health literacy will affect an individual’s health behavior (Zhang et al., 2021).

At present, the research on residents and self-medication in China mostly investigates residents’ knowledge, attitude and behavior of self-medication, and the research on influencing factors mostly involves some surveys on demographic sociology. This study not only investigates the current situation of self-medication among residents in China, but also explores the correlation between factors such as demographic sociology, personality, health literacy and self-assessed health status, etc., and the importance of residents’ perception of drug efficacy and safety. In addition, the previous studies in China are mostly surveys from a small area (such as provinces and cities) and a small sample, and the research results can only represent the residents in this area. The purpose of this study is to carry out a cross-sectional study of large samples covering all provinces of China in China, and the results can represent the overall situation of China. The research results can better support the improvement of rational self-medication behavior of residents in China, make healthy decisions, and provide reference for residents’ health management.

The objective of this study is to investigate the current status of Chinese residents’ self-medication behaviors (including residents’ self-medication rate and the types of drugs they purchase and use), important factors to consider when purchasing OTC drugs (including drug efficacy, drug safety, recommendations from medical staff, family members, friends, etc.) and to explore the relationship between the residents’ demographic and sociological characteristics and their view of drug efficacy and safety as important considerations when purchasing OTC drugs.

## 2 Methods

### 2.1 Study design

The study was conducted in mainland China from 10 July 2021 to 15 September 2021, using a multi-stage sampling method. The specific research design is described in our group’s previously published paper (Zhang Z et al., 2022).

### 2.2 Participants

#### 2.2.1 Calculation of minimum sample size

We used the following formula to calculate the minimum sample size (Samo et al., 2022).

$$n=\left[Z_{\alpha /2}^{2}pq\right]/\delta$$

In the above formula, n represents the sample size, p represents the estimated self-medication rate, q = 1-p, *α* = 0.05, **Z**_**α/2**_ = 1.96 ˜ 2, *δ* is the allowable error, *δ* = 0.1*p. According to literature reports, the self-medication rate of people around the world is about 32.5%–81.5% (Kassie et al., 2018), the smaller value is used for sample size calculation, and the minimum sample size calculated by substituting the formula is 831. Considering 20% of invalid questionnaires, the minimum number of questionnaires that should be distributed is 1039.

#### 2.2.2 Inclusion criteria

(1) Age>18; (2) Had the nationality of the People’s Republic of China; (3) China’s permanent resident population with the annual travel time≤1 month; (4) Participate in the study and fill in the informed consent form voluntarily; (5) Participants can complete the network questionnaire survey by themselves or with the help of investigators; (6) Participants can understand the meaning of each item in the questionnaire; (7) Participants who have self-medicated behavior, in other words, the participant must have purchased and used OTC drugs on their own.

#### 2.2.3 Exclusion criteria

(1) Persons with unconsciousness, or mental disorders; (2) Those who are participating in other similar research projects. (3) Medical staff. (Medical staff has relatively specialized knowledge of medicines, and the objective of this study was to study the self-medication behavior of residents, so medical staff was excluded from this study.)

**FIGURE 1:**
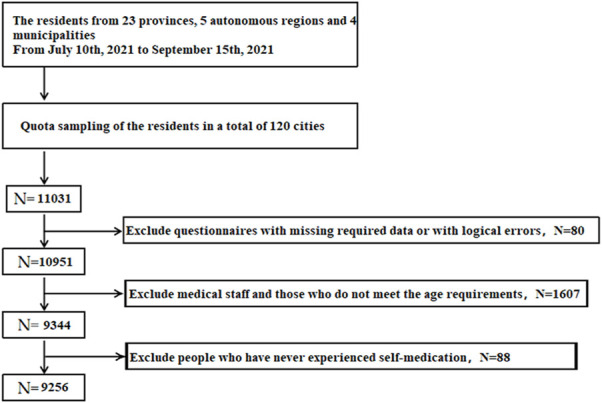
Flowchart of participant enrollment.

Initially, 11,031 participants from 120 cities in mainland China finished the questionnaire. After excluding questionnaires that do not meet the requirements of the study, 9256 residents were enrolled in this study. Figure 1 shows a detailed flowchart of the enrollment. The effective rate of the investigation reached 83.91%.

### 2.3 Instruments

The questionnaire consists of three parts focusing on the current status of residents’ self-medication behaviors and related influencing factors. The first part investigated the social-demographic characteristics of the residents, such as gender, age, province, place of permanent residence (urban, rural), education level, *per capita* monthly income of the family, marital status, the current main way of bearing medical expenses, current occupational status (student, on-the-job, no fixed occupation or retired), currently diagnosed chronic diseases, etc. The second part investigates the current status of residents’ self-medication behaviors and important considerations, including 3 questions (1 single-choice question, and 2 multiple-choice questions). The third part is a series of standard scales, including the 10-item short version of the Big Five Inventory (BFI-10), the Short-Form Health Literacy Instrument (HLS-SF12), and the EQ-5D visual analogue scale (EQ-VAS). Permission was obtained from the developers of HLS-SF12, while the other two scales are available for free for non-commercial use (Duong, T. V et al., 2019; EQ-5D-5L Scale, 2021; Rammstedt and John, 2007; Carciofo et al., 2016).

#### 2.3.1 Items for resident self-medication status and important considerations

This section includes three entries (1 single-choice question, and 2 multiple-choice questions). All entries in this section were designed based on the current sales of OTC drugs in the Chinese market, relevant literature and personal practical experience, combined with expert consultation (Brabers et al., 2013; Barrenberg and Garbe, 2015; Hedenrud et al., 2019; YAN et al., 2019; Watanabe, 2020; Sánchez-Sánchez et al., 2021; El-Gamal et al., 2022). The expert group consists of eight pharmacists with bachelor’s degrees or above working in secondary or tertiary hospitals.

The single-choice question is “Have you ever purchased and used OTC medicines on your own?”. Respondents who answered “No” to this question were excluded from the study.

The first multiple-choice question is “What kinds of OTC drugs have you ever purchased and used? “, this question has 10 options, namely, (1) Antipyretic analgesics (e.g.,: paracetamol); (2) Digestive system drugs (e.g.,: ranitidine hydrochloride capsules); (3) Respiratory system drugs (e.g.,: aminocaffeine tablets); (4) Vitamins/minerals (e.g.,: vitamin C tablets); (5) Antibacterial drugs (e.g.,: metronidazole buccal tablets); (6) Drugs for external use (e.g.,: compound beclomethasone camphor cream); (7) Chinese patent drugs (e.g.,: Xiao Chai Hu granules, Xiao Jianzhong granules, Sijunzi pills); (8)Gynecological drugs (e.g.,: miconazole nitrate suppositories); (9) Anti-allergic drugs (e.g.,: loratadine capsules); (10) Others.

The second multiple-choice question is “Which of the following factors are important considerations when purchasing OTC drugs?”. The 16 options for this question are: (1) The price of the drug; (2) The efficacy of the drug; (3) The safety of the drug; (4) The taste of the drug; (5) Brand awareness; (6) Whether the drug can be reimbursed by medical insurance; (7) Advice from medical staff (including doctors, pharmacists, etc.); (8) Advice from family members; (9) Advice from friends; (10) Personal experience; (11) Advertising; (12) After-sales service; (13) Corporate reputation; (14) Ease of taking the drug; (15) Packaging of the drug; (16) Dosage form of the drug. The order in which the options appear in the two multiple-choice questions is random for each respondent.

#### 2.3.2 The 10-item short version of the Big Five Inventory (BFI-10)

The 10-item short version of the Big Five Inventory (BFI-10) was applied to measure the personality characteristics of residents, including Extraversion, Agreeableness, Conscientiousness, Neuroticism, and Openness, on a 5-point Likert-type scale ranging from 1 (totally disagree) to 5 (totally agree) (Rammstedt and John, 2007; Carciofo et al., 2016). The scores of Extraversion were summed of the scores of item 1R and item 6, the scores of Agreeableness were combined with the scores of item 2 and 7R, Conscientiousness as 3R and 8, Neuroticism as 4R + 9, and Openness as 5R + 10 (R = item is reversed-scored). Several studies have shown that BFI-10 has good reliability and validity (Johann et al., 2020; Eichenberg et al., 2021; Nikčević et al., 2021). In a previous study, the reliability levels of the BFI-10 proved satisfactory using Cronbach’s *α* analysis: Extraversion (*α* = 0.723), Agreeableness (*α* = 0.759), Conscientiousness (*α* = 0.786), Neuroticism (*α* = 0.753) and Openness to experience (*α* = 0.714) (Wang et al., 2014). The higher the score of a personality trait of the respondent, the more significant the personality trait of the respondent is. In this study, referring to relevant literature, the five personality characteristics of the respondents were divided into a high group (7–10 points) and a low group (6 points and below) (Heilmann et al., 2021).

#### 2.3.3 The short-form health literacy instrument (HLS-SF12)

The health literacy of the respondents was measured by HLS-SF12 (Duong, T. V et al., 2019). The scale includes 3 dimensions of healthcare, disease prevention, and health promotion, with a total of 12 items, and each item is scored on a 4-point scale (1 = very difficult, 2 = difficult, 3 = easy, 4 = very easy). In the study, the Cronbach’s coefficient of the scale was 0.940, and the Cronbach’s coefficients of the three subscales of healthcare, disease prevention and health promotion were 0.856, 0.860, and 0.868, respectively, with good reliability. The higher the respondent’s score on this scale, the higher the health literacy of the respondent. In this study, referring to relevant literature, the health literacy of the surveyed subjects was divided into a high group (over 33 points) and a low group (33 points and below) (The European Health Literacy Project 2009–2012; Cheong et al., 2021).

#### 2.3.4 The EQ-5D visual analogue scale

The European Five-dimensional Health Scale (EQ-5D-5L) is one of the most widely used health-related quality of life measurement tools to measure the health status of the population (Jiang et al., 2021). EQ-VAS is a part of EQ-5D-5L. Respondents filled in integers between 0 and 100 to indicate their health status. 100 represents the respondent’s best-imagined health status, while 0 represents the respondent’s worst-imagined health status (Ping et al., 2020). In this study, referring to relevant literature, the EQ-VAS scores of the respondents were divided into a high group (81–100 points) and a low group (80 points and below) (Wang et al., 2017).

### 2.4 Statistical methods

Data entry and analysis were performed using SPSS™ for Windows (version 27.0) (SPSS Inc, Chicago, IL, United States of America). First, the common method bias was tested. Then the quantity and percentage of categorical variables were calculated using descriptive statistics. Scale scores were tested for normality. For normally distributed data, the mean and standard deviation were used for statistical description, and non-normally distributed data, the median and interquartile range were used for statistical description. Regarding the relevant literature, all scale scores were converted into dichotomous variables (high grouping and low grouping). The independent variables in the study include the demographic and sociological characteristics, health literacy, personality, and self-assessed health status of the respondents. There are two dependent variables, which are whether the respondents consider drug efficacy or safety as an important factor when purchasing OTC drugs. The Chi-square test was used for univariate analysis. In the study, the percentage of respondents who cited drug efficacy or safety as an important consideration in purchasing OTC drugs both exceeded fifty percent. In this case, using traditional logistic regression for multifactor analysis may overestimate the association between the independent and dependent variables; therefore, multilevel log-bionmial regression was used for multifactor analysis to overcome the level effects (Barros and Hirakata, 2003; Deddens and Petersen, 2008; Knol et al., 2012; Marschner and Gillett, 2012), and conduct stratified analysis according to different locations (Eastern part of China, Central part of China and Western part of China). Unless otherwise stated, the test level of statistical tests was *α* = 0.05. A supplementary subgroup analysis of demographic characteristics was performed, and subgroups were divided according to gender (male, female), age (35 years old and below, above 35 years old), permanent residence (urban, rural) in the proportion of residents considering efficacy or safety in different regions of China are highlighted in the paper.

### 2.5 Quality control

The study conducted two rounds of pre-investigation and two rounds of expert consultation before the formal survey. Trained investigators distributed questionnaires to respondents and registered their codes one-on-one and face-to-face. Every Sunday evening during the investigation process, members of the research group communicated with the investigators to summarize, evaluate, and give feedback on the questionnaires they collected. After the questionnaires were collected, two people conducted back-to-back logic checks and data screening. If singular values are found during data analysis, the original questionnaire must be found and checked with the investigator before proceeding to the next step of the analysis.

## 3 Results

### 3.1 Common method bias test

Harman’s single-factor method showed five factors with eigenvalues greater than 1, and the variance contribution rate of the first main factor was 34.98%, which did not exceed 40%, indicating that there was no common method bias (Podsakoff et al., 2003).

### 3.2 Demographic and sociological characteristics of the respondents

The investigation showed that 99.06% (9256 of 9344) of Chinese people aged 18 years or older had self-medication behaviors.

Among respondents 9256 who had self-medication behaviors, there were 4289 males (46.3%) and 4967 females (53.7%) among the respondents; 4722 (51.0%) were located in eastern China, 2391 (25.8%) were located in central China, and 2143 (23.2%) were located in western China; 6674 (72.1%) were urban residents, 2582 (27.9%) were rural; 4246 (45.9%) were 19–35 years old, and 3935 (42.5%) were 36–59 years old, 1075 (11.6%) were aged 60 and above. The demographic and sociological characteristics of the survey respondents are shown in Table 1.

**TABLE 1:** Demographic and sociological characteristics of respondents.

Variables	Number	Percentage (%)
Gender		
Male	4289	46.3
Female	4967	53.67
Age(years)		
19–35	4246	45.9
36–59	3935	42.5
≥60	1075	11.6
Education level		
High/Secondary School and lower	3685	39.8
Junior college	1300	14.0
Undergraduate	3654	39.5
Postgraduate degree (including Masters and Ph.D. students)	617	6.7
Location		
Eastern part of China	4722	51.0
Central part of China	2391	25.8
Western part of China	2143	23.2
The main way of medical expenses borne		
Out-of-pocket Payment	1840	19.9
Resident Basic Medical Insurance (RBMI)	4472	48.3
Others (Basic medical insurance for employees, Commercial medical insurance, Free medical treatment)	2944	31.8
Place of residence		
Urban	6674	72.1
Rural	2582	27.9
Monthly income (RMB)		
0–4500 (0$-666$)	4735	51.2
4501–9000 (666.148$-1332$)	3146	34.0
>9000 (1332$)	1375	14.9
Marital Status		
Unmarried	5765	62.3
Married	3072	33.2
Divorced	193	2.1
Widowed	226	2.4
Employment status		
Employed	4129	44.6
Student	2144	23.2
Unemployed	2174	23.5
Retired	809	8.7
Chronic diseases condition		
No chronic diseases	7357	79.5
Hypertension	1051	11.4
Diabetes Mellitus	243	2.6
Dyslipidemia	285	3.1
Coronary atherosclerotic heart disease	211	2.3
Chronic gastritis	314	3.4
Fatty liver disease	106	1.2
Chronic enteritis	89	1.0
Asthma	60	0.7
Chronic nephritis	42	0.5
Cerebral apoplexy (Cerebral infarction, cerebral hemorrhage, etc.)	71	0.8
Malignant tumor	40	0.4
Viral hepatitis	22	0.2
Chronic obstructive pulmonary diseases	15	0.2
Parkinson’s disease	18	0.2

### 3.3 Status of self-medication of the respondents

**FIGURE 2:**
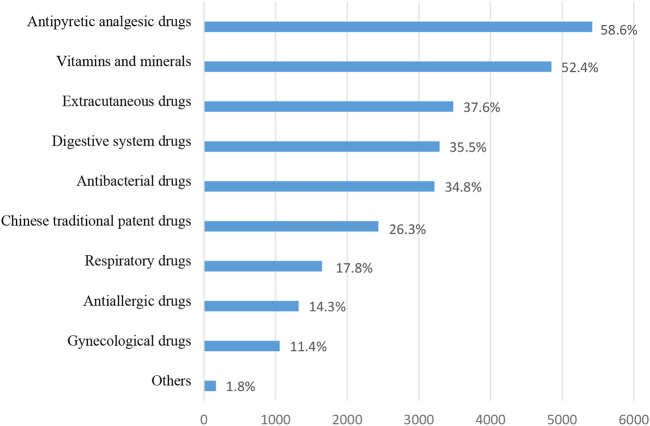
Types of OTC drugs purchased by respondents.

Among respondents 9256 who had self-medication behaviors, the investigation showed that 99.1% (9256 of 9344) of Chinese people aged 18 years or older had self-medication behaviors. The types of drugs used by the respondents during self-medication are shown in Figure 2. Antipyretic analgesics (5421, 58.6%) and vitamins/minerals (4851, 52.4%) ranked the top two among all types of drugs. In addition to the “other” option, the number of users of gynecological drugs (1057, 11.4%) and anti-allergy drugs (1322, 14.3%) ranked the bottom two.

### 3.4 Important considerations for respondents when purchasing OTC drugs

**FIGURE 3:**
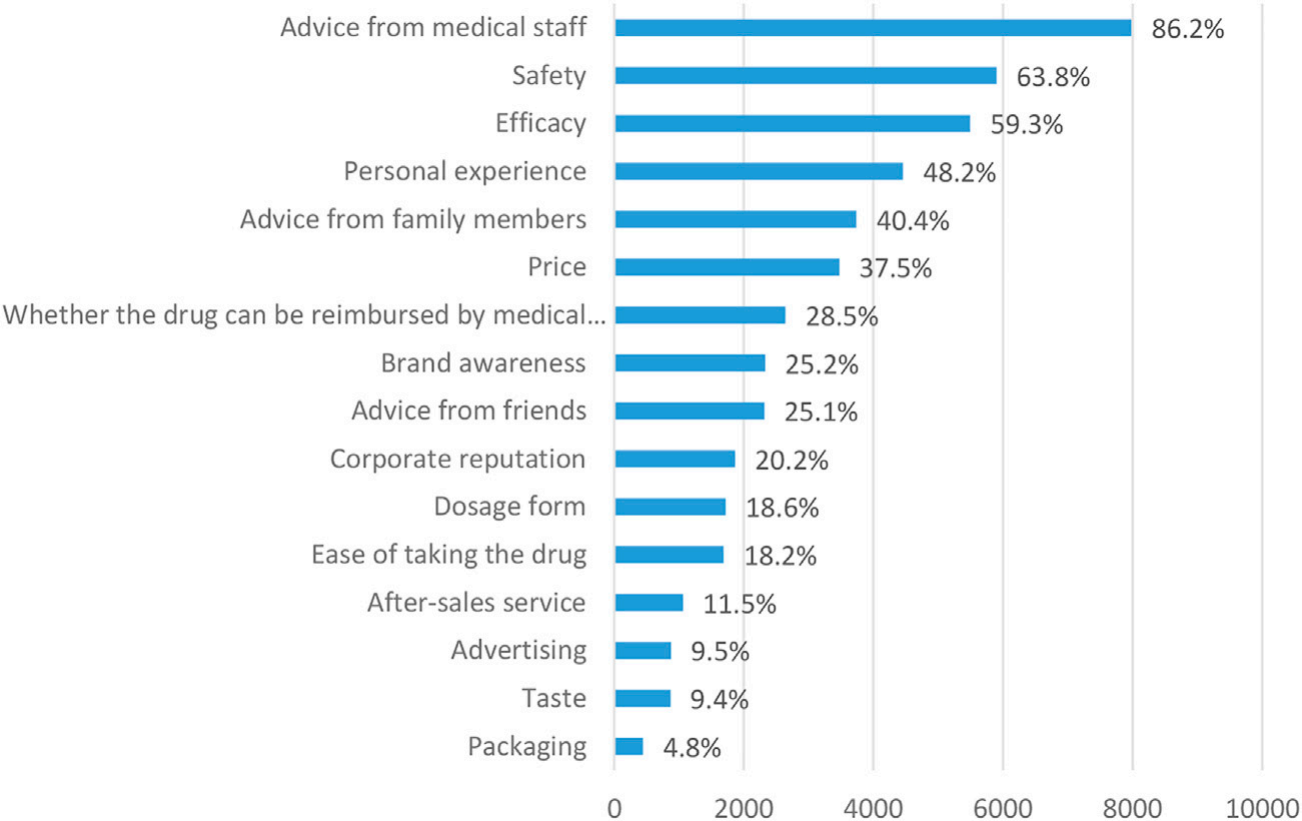
Important considerations when respondents purchase OTC drugs.

Among the 16 factors to be considered, the top three selected are medical staff advice (7979, 86.2%), drug safety (5901, 63.7%) and drug efficacy (5492, 59.3%), the last three choices were exquisiteness of medicine packaging (445 people, 4.8%), the taste of medicines (871 people, 9.4%) and advertising (878 people, 9.5%) (Figure 3).

### 3.5 The scores of each scale of the respondents

The scores of the respondents on each scale are shown in Table 2. Since the scores on each scale do not satisfy the normal distribution, the median and upper and lower quartiles were used to describe the central tendency and dispersion of the scores of each scale. 5942 respondents (64.2%) had high health literacy; There are 3349 people (36.2%) with high extroversion, 5182 people (56.0%) with high agreeableness, 4757 people (51.4%) with high conscientiousness, 2257 people (24.4%)with high neuroticism, 3565 people (38.5%)with high openness; and 5460 people (59.0%) with high self-rated health status.

**TABLE 2:** Respondents’ scores on HLS-SF12, BFI-10 and EQ-VAS.

	No. of items	Score range	Kolmogorow-smironov Z	*p*-Value from K-S test	Median	Q1-Q3	No. and percentage of high score group	No. and percentage of low score group
**HLS—SF12**	12	0–50	0.208	<0.001	33.33	30.56–37.50	5942 (64.2%)	3314 (35.8%)
**BFI-10**								
Extraversion	2	2–10	0.203	<0.001	6	5–7	3349 (36.2%)	5907 (63.8%)
Agreeableness	2	2–10	0.187	<0.001	7	6–8	5182 (56.0%)	4074 (44.0%)
Conscientiousness	2	2–10	0.200	<0.001	7	6–8	4757 (51.4%)	4499 (48.6%)
Neuroticism	2	2–10	0.217	<0.001	6	5–6	2257 (24.4%)	6999 (75.6%)
Openness	2	2–10	0.221	<0.001	6	5–7	3565 (38.5%)	5691 (61.5%)
**EQ-VAS**	1	0–100	0.148	<0.001	84	73–96	5460 (59.0%)	3796 (41.0%)

### 3.6 Chi-square test results of two factors of drug efficacy and safety

Univariate analysis was performed using the chi-square test for the likelihood that respondents considered drug efficacy or safety as an important consideration. The possibility of respondents taking drug safety as an important consideration varies in gender, age, region, mode of paying medical expenses, chronic disease, extraversion, agreeableness, conscientiousness, neuroticism, openness, health literacy and self-assessed health status (*p <* 0.05). The possibility of respondents taking drug efficacy as an important consideration varies in gender, age, region, marital status, occupational status, chronic disease, extraversion, agreeableness, conscientiousness, openness, health literacy, and self-assessed health status (*p <* 0.05). The results of the chi-square test are shown in Supplementary Tables S1, S2.

### 3.7 Differences in the percentage of residents who focus on safety or efficacy of self-medication in different regions of China

**FIGURE 4:**
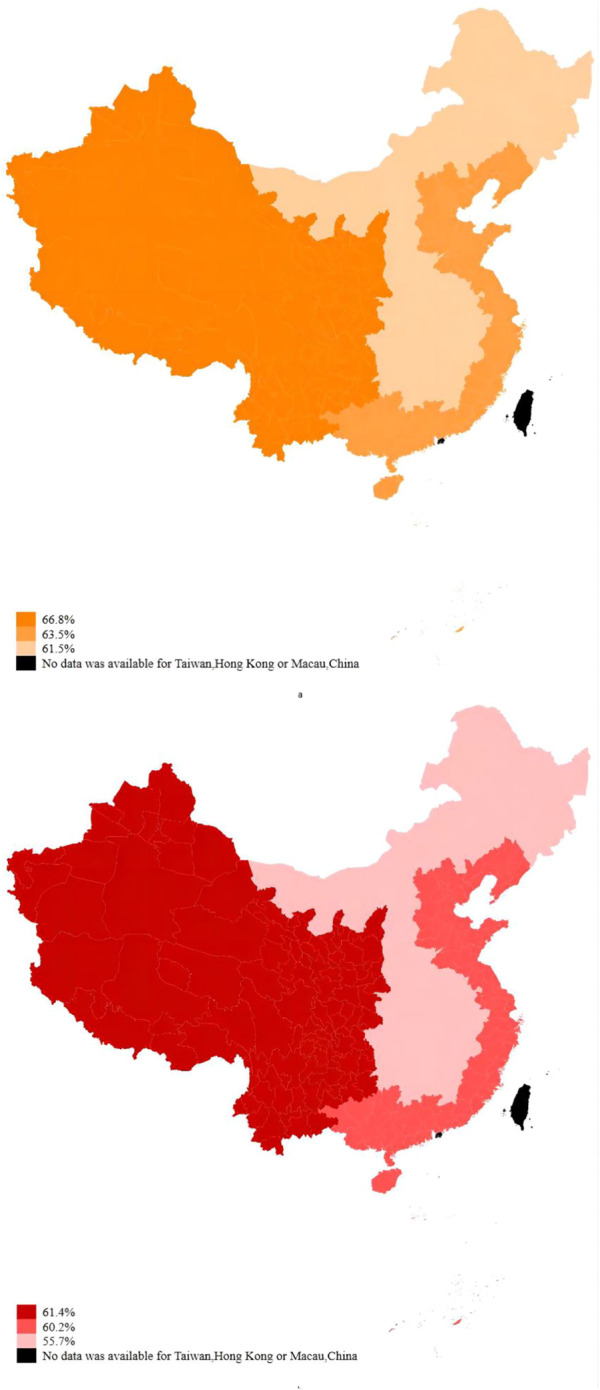
Distribution of residents focusing on safety or efficacy in different regions of China (a:safety; b:efficacy).

A chi-square test was used to conduct a single factor analysis of differences in the percentage of residents who focused on safety or efficacy when self-medicating. The results of the chi-square test are shown in Supplementary Tables S1, S2. The results showed that the distribution of residents who focused on safety o refficacy differed across different regions of China (east, central, and west) (Safety:*p* = 0.001; Efficacy: *p* < 0.001). Multiple comparisons were further performed by the Bonferroni method, and it is shown that a significantly higher percentage of residents in the west (66.8%) focus on safety than those in the east (63.5%) and central (61.5%) regions. As for efficacy, the proportion of residents in the central region (55.7%) who focused on efficacy was significantly lower than those in the eastern (60.2%) and western regions (61.4%) (See Figure 4 for details).

### 3.8 Multivariate regression analysis of two factors of drug efficacy and safety

#### 3.8.1 Drug safety

Multilevel log-binomial regression analysis was carried out with the possibility of the respondents considering drug safety as an important factor as the dependent variable, the demographic and sociological characteristics of the respondents and the grading of each scale score as the independent variables. Three regression models were developed, the first regression model with respondents’ demographic and sociological characteristics as independent variables, the second regression model with respondents’ scale score grading as independent variables, and the third regression model with respondents’ demographic and sociological characteristics and scale score grading as independent variables. Using the third model as the main result of this study, the multilevel regression results showed that model 3 was robust.

The Omnibus test result of the model 3 is *p* < 0.001, the log-likelihood value is −5515.351, indicating that the model is of good quality. Log-binomial regression analysis showed that gender, location, employment status, the main way of medical expenses borne, agreeableness, conscientiousness, neuroticism, openness, health literacy, and self-rated health status were related to whether respondents considered drug safety as an important consideration when purchasing OTC drugs. Compared with men, women were more likely to take drug safety as an important consideration (PRR < Percentage rate ratio ≥1.052, 95%CI 1.021–1.085, *p* = 0.001).

Compared with the respondents in the east, the respondents in western parts were more likely to consider drug safety as an important factor (PRR = 1.044, 95%CI 1.007–1.082, *p* = 0.020); Compared with the respondents who were employed, retired people were more likely to consider drug safety as an important factor (PRR = 1.090, 95%CI 1.020–1.166, *p* = 0.012); Compared with the respondents whose main way of medical expenses borne was Out-of-pocket Payment, those who mainly used ways including basic medical insurance for employees, commercial medical insurance, free medical treatment to cover their medical cost were more likely to consider drug safety as an important factor (PRR = 1.053, 95%CI 1.002–1.106, *p* = 0.040). Compared with high agreeableness respondents, low agreeableness respondents were less likely to consider drug safety as an important consideration (PRR = 0.896, 95%CI 0.865–0.927, *p* < 0.001); Compared with high conscientiousness respondents, low conscientiousness respondents were less likely to consider drug safety as an important consideration (PRR = 0.931, 95%CI 0.900–0.963, *p* < 0.001); Compared with those with high neuroticism, respondents with low neuroticism were less likely to consider drug safety as an important consideration (PRR = 0.959, 95%CI 0.928–0.992, *p* = 0.014); Compared with those with high openness, those with low openness were less likely to consider drug safety as an important consideration (PRR = 0.941, 95%CI 0.911–0.972, *p <* 0.001); Compared with respondents with high health literacy, respondents with low health literacy were less likely to consider drug safety as an important factor (PRR = 0.960, 95%CI 0.927–0.993, *p* = 0.018); Compared with respondents with better self-rated health status, respondents with poorer self-rated health status were less likely to consider drug safety as an important consideration (PRR = 0.915, 95%CI 0.885–0.945, *p* < 0.001) (See Table 3 for details). Subgroup analysis was carried out according to gender, age, and place of permanent residence, and a total of six models were established. The independent variables and model parameters of the six subgroup analysis models were similar to the model built by all respondents. (See Supplementary Tables S3–S8 for details.

**TABLE 3:** Multilevel log-binomial regression results with drug safety as the dependent variable

Models	Variables	β	SE	*Wald χ* ^*2*^	*p*	PRR	The lower limit of 95%*CI*	The upper limit of 95%*CI*
**Model 1**	**Gender** (**control group = Male)**							
	**Female**	0.073	0.0159	21.135	<0.001	1.076	1.043	1.110
	**Age (control group = 19–35)**							
	**36–59**	0.046	0.0234	3.916	0.048	1.047	1.000	1.097
	**60 or above**	−0.071	0.0409	3.038	0.081	0.931	0.860	1.009
	**Educational level (control group = High/Secondary School and lower)**							
	**Junior college**	0.009	0.0255	0.132	0.716	1.009	0.960	1.061
	**Undergraduate**	0.007	0.0228	0.101	0.750	1.007	0.963	1.053
	**Postgraduate**	−0.048	0.0380	1.586	0.208	0.953	0.885	1.027
	**Location (control group = Eastern part of China)**							
	**Central part of China**	−0.024	0.0197	1.454	0.228	0.977	0.940	1.015
	**Western part of China**	0.057	0.0189	9.106	0.003	1.059	1.020	1.099
	**Place of residence (control group = Rural)**							
	**Urban**	−0.026	0.0189	1.851	0.174	0.975	0.939	1.011
	**Marital Status (control group = Unmarried)**							
	**Married**	0.010	0.0285	0.129	0.720	1.010	0.955	1.068
	**Divorced**	−0.107	0.0608	3.070	0.080	0.899	0.798	1.013
	**Widowed**	−0.064	0.0615	1.070	0.301	0.938	0.832	1.059
	**Employment status (control group = Employed)**							
	**Student**	0.036	0.0307	1.378	0.240	1.037	0.976	1.101
	**Unemployed**	0.006	0.0241	0.054	0.816	1.006	0.959	1.054
	**Retired**	0.077	0.0347	4.988	0.026	1.080	1.010	1.156
	**The main way of medical expenses borne (control group = Out-of-pocket Payment)**							
	**Resident Basic Medical Insurance (RBMI)**	0.008	0.0218	0.141	0.707	1.008	0.966	1.052
	**Others (Basic medical insurance for employees, Commercial medical insurance, Free medical treatment)**	0.080	0.0259	9.595	0.002	1.083	1.030	1.140
	**Chronic diseases condition (control group = No chronic diseases)**							
	**Suffer from chronic diseases**	−0.037	0.0221	2.751	0.097	0.964	0.923	1.007
	**Monthly income (RMB) (control group = 0–4500 (0$-666$))**							
	**4501–9000 (666.148$-1332$)**	−0.010	0.0182	0.323	0.570	0.990	0.955	1.026
	**>9000 (1332$)**	0.008	0.0246	0.105	0.746	1.008	0.961	1.058
**Model 2**	**Extraversion (control group = High score group)**							
	**Low score group**	−0.024	0.0162	2.169	0.141	0.976	0.946	1.008
	**Agreeableness (control group = High score group)**							
	**Low score group**	−0.119	0.0175	46.069	<0.001	0.888	0.858	0.919
	**Conscientiousness (control group = High score group)**							
	**Low score group**	−0.075	0.0167	20.055	<0.001	0.928	0.898	0.959
	**Neuroticism (control group = High score group)**							
	**Low score group**	−0.049	0.0172	8.144	0.004	0.952	0.921	0.985
	**Openness (control group = High score group)**							
	**Low score group**	−0.059	0.0162	13.401	<0.001	0.942	0.913	0.973
	**Health Literacy (control group = High score group)**							
	**Low score group**	−0.041	0.0170	5.911	0.015	0.959	0.928	0.992
	**EQ-VAS (control group = High score group)**							
	**Low score group**	−0.095	0.0168	32.460	<0.001	0.909	0.880	0.939
**Model 3**	**Gender (control group = Male)**							
	**Female**	0.051	0.0156	10.704	0.001	1.052	1.021	1.085
	Age (control group = 19–35)							
	36–59	0.036	0.0231	2.360	0.124	1.036	0.990	1.084
	60 or above	−0.072	0.0409	3.128	0.077	0.930	0.859	1.008
	**Educational level (control group = High/Secondary School and lower)**							
	Junior college	0.018	0.0245	0.512	0.474	1.018	0.970	1.068
	Undergraduate	−0.012	0.0222	0.270	0.603	0.989	0.947	1.032
	Postgraduate	−0.060	0.0373	2.584	0.108	0.942	0.875	1.013
	**Location (control group = Eastern part of China)**							
	Central part of China	−0.029	0.0193	2.252	0.133	0.971	0.935	1.009
	Western part of China	0.043	0.0184	5.424	0.020	1.044	1.007	1.082
	**Place of residence (control group = Rural)**							
	Urban	−0.030	0.0184	2.590	0.108	0.971	0.936	1.006
	**Marital Status (control group = Unmarried)**							
	Married	0.019	0.0279	0.477	0.49	1.019	0.965	1.077
	Divorced	−0.100	0.0592	2.834	0.092	0.905	0.806	1.017
	Widowed	−0.05	0.0615	0.664	0.415	0.951	0.843	1.073
	**Employment status (control group = Employed)**							
	Student	0.027	0.0297	0.807	0.369	1.027	0.969	1.088
	Unemployed	0.03	0.0236	1.62	0.203	1.031	0.984	1.079
	Retired	0.086	0.0342	6.381	0.012	1.09	1.02	1.166
	**The main way of medical expenses borne (control group = Out-of-pocket Payment)**							
	Resident Basic Medical Insurance (RBMI)	−0.007	0.0212	0.112	0.738	0.993	0.953	1.035
	Others (Basic medical insurance for employees, Commercial medical insurance, Free medical treatment)	0.052	0.0252	4.226	0.04	1.053	1.002	1.106
	**Chronic diseases condition (control group = No chronic diseases)**							
	Suffer from chronic diseases	−0.014	0.0218	0.414	0.52	0.986	0.945	1.029
	**Monthly income (RMB) (control group = 0–4500(0$-666$))**							
	4501–9000(666.148$-1332$)	−0.017	0.0177	0.916	0.339	0.983	0.95	1.018
	>9000(1332$)	0.005	0.024	0.035	0.851	1.005	0.958	1.053
	**Extraversion (control group = High score group)**							
	Low score group	−0.027	0.0161	2.891	0.089	0.973	0.943	1.004
	**Agreeableness (control group = High score group)**							
	Low score group	−0.11	0.0175	39.581	<0.001	0.896	0.865	0.927
	**Conscientiousness (control group = High score group)**							
	Low score group	−0.071	0.0172	17.071	<0.001	0.931	0.9	0.963
	**Neuroticism (control group = High score group)**							
	Low score group	−0.042	0.0171	6.002	0.014	0.959	0.928	0.992
	**Openness (control group = High score group)**							
	Low score group	−0.061	0.0165	13.555	<0.001	0.941	0.911	0.972
	**Health Literacy (control group = High score group)**							
	Low score group	−0.041	0.0175	5.57	0.018	0.96	0.927	0.993
	**EQ-VAS (control group = High score group)**							
	Low score group	−0.089	0.0169	27.766	<0.001	0.915	0.885	0.945

The number in bold is *p*<0.05.

#### 3.8.2 Drug efficacy

Multilevel log-binomial regression analysis was carried out with the possibility of the respondents taking drug efficacy as an important consideration factor as the dependent variable, and the demographic and sociological characteristics of the respondents and the grading of each scale score as the independent variables. Three regression models were developed, the model 4 with respondents’ demographic and sociological characteristics as independent variables, the model 5 with respondents’ scale score grading as independent variables, and the model 6 with respondents’ demographic and sociological characteristics and scale score grading as independent variables. Using the third model as the main result of this study, the multilevel regression results showed that model six was robust.

The Ominbus test result of the model six is *p* < 0.001, the log-likelihood value is −5689.403, indicating that the model is of good quality. Log-binomial regression analysis showed that gender, location, employment status, the main way of medical expenses borne, chronic disease, agreeableness, conscientiousness, openness, and self-rated health status were related to whether respondents considered drug efficacy as an important consideration when purchasing OTC drugs. Compared with men, women were more likely to consider drug efficacy as an important factor (PRR = 1.056, 95%CI 1.021–1.092, *p* = 0.001); Compared with eastern respondents, midlands respondents were less likely to consider drug efficacy as an important factor (PRR = 0.916, 95%CI 0.878–0.956, *p <* 0.001); Compared with the respondents who were employed, unemployed people were less likely to consider drug efficacy as an important factor (PRR = 0.939, 95%CI 0.890–0.990, *p* = 0.019); Compared with the respondents whose main way of medical expenses borne was Out-of-pocket Payment, those who mainly used Resident Basic Medical Insurance to cover their medical cost were less likely to consider drug efficacy as an important factor (PRR = 0.946, 95%CI 0.904–0.989, *p* = 0.015). Compared with those without chronic disease, those with chronic disease were more likely to consider drug efficacy as an important factor (PRR = 1.069, 95%CI 1.022–1.118, *p* = 0.004); Compared with high agreeableness respondents, low agreeableness respondents were less likely to consider drug efficacy as an important factor (PRR = 0.900, 95%CI 0.867–0.935, *p* < 0.001); Compared with high conscientiousness respondents, low conscientiousness respondents were less likely to consider drug efficacy as an important factor (PRR = 0.881, 95%CI 0.848–0.915, *p* < 0.001); Compared with high openness respondents, low openness respondents were less likely to consider drug efficacy as an important factor (PRR = 0.953, 95%CI 0.920–0.988, *p* = 0.009); Compared with respondents with better self-rated health status, respondents with poor self-rated health status were less likely to consider drug efficacy as an important consideration (PRR = 0.916 95%CI 0.883–0.950, *p* < 0.001) (See Table 4 for details). Subgroup analysis was carried out according to gender, age and place of permanent residence, and a total of six models were established. The independent variables and model parameters of the six subgroup analysis models were similar to the model built by all respondents. (See Supplementary Tables S9–S14 for details).

**TABLE 4:** Multilevel log-binomial regression results with drug efficacy as the dependent variable.

Models	Variables	β	SE	*Wald χ* ^*2*^	*p*	PRR	The lower limit of 95%*CI*	The upper limit of 95%*CI*
**Model 4**	**Gender (control group =Male)**							
	**Female**	0.072	0.0175	16.784	**<0.001**	1.074	1.038	1.112
	**Age (control group =19–35)**							
	**36–59**	0.034	0.0256	1.808	0.179	1.035	0.984	1.088
	**60 or above**	0.022	0.0440	0.259	0.611	1.023	0.938	1.115
	**Educational level (control group =High/Secondary School and lower)**							
	**Junior college**	0.035	0.0285	1.479	0.224	1.035	0.979	1.095
	**Undergraduate**	0.036	0.0254	1.972	0.160	1.036	0.986	1.089
	**Postgraduate**	0.002	0.0411	0.003	0.959	1.002	0.925	1.086
	**Location (control group =Eastern part of China)**							
	**Central part of China**	−0.078	0.0219	12.740	**<0.001**	0.925	0.886	0.965
	**Western part of China**	0.023	0.0210	1.235	0.266	1.024	0.982	1.066
	**Place of residence (control group =Rural)**							
	**Urban**	−0.026	0.0209	1.552	0.213	0.974	0.935	1.015
	**Marital Status (control group =Unmarried)**							
	**Married**	−0.042	0.0318	1.726	0.189	0.959	0.901	1.021
	**Divorced**	−0.097	0.0653	2.214	0.137	0.907	0.799	1.031
	**Widowed**	0.002	0.0576	0.001	0.978	1.002	0.895	1.121
	**Employment status (control group =Employed)**							
	**Student**	−0.005	0.0348	0.024	0.877	0.995	0.929	1.065
	**Unemployed**	−0.080	0.0275	8.469	**0.004**	0.923	0.875	0.974
	**Retired**	0.002	0.0576	0.001	0.978	1.002	0.895	1.121
	**The main way of medical expenses borne (control group =Out-of-pocket Payment)**							
	**Resident Basic Medical Insurance (RBMI)**	−0.030	0.0234	1.651	0.199	0.970	0.927	1.016
	**Others (Basic medical insurance for employees, Commercial medical insurance, Free medical treatment)**	−0.011	0.0282	0.150	0.699	0.989	0.936	1.045
	**Chronic diseases condition (control group =No chronic diseases)**							
	**Suffer from chronic diseases**	0.047	0.0234	3.981	**0.046**	1.048	1.001	1.097
	**Monthly income (RMB) (control group =0–4500 (0$-666$))**							
	**4501–9000 (666.148$-1332$)**	−0.022	0.0199	1.223	0.269	0.978	0.941	1.017
	**>9000 (1332$)**	−0.044	0.0276	2.567	0.109	0.957	0.906	1.010
**Model 5**	**Extraversion (control group =High score group)**							
	**Low score group**	−0.004	0.0179	0.050	0.824	0.996	0.962	1.032
	**Agreeableness (control group =High score group)**							
	**Low score group**	−0.110	0.0192	32.892	**<0.001**	0.896	0.863	0.930
	**Conscientiousness (control group =High score group)**							
	**Low score group**	−0.136	0.0188	52.354	**<0.001**	0.873	0.841	0.906
	**Neuroticism (control group =High score group)**							
	**Low score group**	−0.014	0.0195	0.509	0.476	0.986	0.949	1.025
	**Openness (control group =High score group)**							
	**Low score group**	−0.046	0.0179	6.665	**0.010**	0.955	0.922	0.989
	**Health Literacy (control group =High score group)**							
	**Low score group**	−0.009	0.0185	0.232	0.630	0.991	0.956	1.028
	**EQ-VAS (control group =High score group)**							
	**Low score group**	−0.075	0.0185	16.560	**<0.001**	0.928	0.895	0.962
**Model 6**	**Gender (control group =Male)**							
	**Female**	0.055	0.0172	10.183	**0.001**	1.056	1.021	1.092
	Age (control group = 19–35)							
	36–59	0.017	0.0254	0.475	0.491	1.018	0.968	1.07
	60 or above	0.029	0.0434	0.457	0.499	1.03	0.946	1.121
	**Educational level (control group =High/Secondary School and lower)**							
	Junior college	0.036	0.0279	1.665	0.197	1.037	0.982	1.095
	Undergraduate	0.02	0.025	0.626	0.429	1.02	0.971	1.071
	Postgraduate	−0.004	0.0407	0.008	0.928	0.996	0.920	1.079
	**Location (control group =Eastern part of China)**							
	Central part of China	−0.088	0.0215	16.618	<0.001	0.916	0.878	0.956
	Western part of China	0.009	0.0204	0.213	0.645	1.009	0.970	1.051
	**Place of residence (control group =Rural)**							
	Urban	−0.026	0.0203	1.681	0.195	0.974	0.936	1.014
	**Marital Status (control group =Unmarried)**							
	Married	−0.02	0.0314	0.401	0.526	0.98	0.922	1.043
	Divorced	−0.089	0.0638	1.935	0.164	0.915	0.808	1.037
	Widowed	0.012	0.0571	0.041	0.84	1.012	0.905	1.131
	**Employment status (control group =Employed)**							
	Student	−0.018	0.0343	0.289	0.591	0.982	0.918	1.05
	Unemployed	−0.063	0.0271	5.461	**0.019**	0.939	0.89	0.99
	Retired	0.018	0.0378	0.229	0.633	1.018	0.946	1.096
	**The main way of medical expenses borne (control group =Out-of-pocket Payment)**							
	Resident Basic Medical Insurance (RBMI)	−0.056	0.0229	5.891	**0.015**	0.946	0.904	0.989
	Others (Basic medical insurance for employees, Commercial medical insurance, Free medical treatment)	−0.054	0.0278	3.718	0.054	0.948	0.897	1.001
	**Chronic diseases condition (control group =No chronic diseases)**							
	Suffer from chronic diseases	0.067	0.023	8.446	**0.004**	1.069	1.022	1.118
	**Monthly income (RMB) (control group =0–4500(0$-666$))**							
	4501–9000 (666.148$-1332$)	−0.025	0.0194	1.706	0.191	0.975	0.939	1.013
	>9000 (1332$)	−0.049	0.0271	3.269	0.071	0.952	0.903	1.004
	**Extraversion (control group =High score group)**							
	Low score group	−0.008	0.0178	0.193	0.661	0.992	0.958	1.027
	**Agreeableness (control group =High score group)**							
	Low score group	−0.105	0.0192	29.899	<0.001	0.900	0.867	0.935
	**Conscientiousness (control group =High score group)**							
	Low score group	−0.127	0.0194	42.549	<0.001	0.881	0.848	0.915
	**Neuroticism (control group =High score group)**							
	Low score group	−0.011	0.0195	0.331	0.565	0.989	0.952	1.027
	**Openness (control group =High score group)**							
	Low score group	−0.048	0.0181	6.920	**0.009**	0.953	0.920	0.988
	**Health Literacy (control group =High score group)**							
	Low score group	−0.022	0.0191	1.307	0.253	0.978	0.942	1.016
	**EQ-VAS (control group =High score group)**							
	Low score group	−0.088	0.0188	21.896	<0.001	0.916	0.883	0.950

The number in bold is *p*<0.05.

#### 3.8.3 Results of subgroup analysis in different regions

In addition to the above subgroup analysis, we also conducted subgroup analysis by region (eastern China, central China, and western China), adjust factors gender, age, education level, the main way of medical expenses borne, place of residence, monthly income, marital Status, employment status, chronic diseases condition, mainly analyze the influence of the Big Five Inventory (BFI-10) Health Life (HLS-SF12) health-related quality of life (EQ-5D-5L) on the safety and effectiveness of self-medication, and the results are shown in Tables 5, 6. The correlates of the dependent variable vary among residents of different regions.

**TABLE 5:** Results of analysis of subgroups by region (dependent variable: likelihood of residents focusing on safety).

Models	Variables	β	SE	*Wald χ* ^*2*^	*p*	PRR	The lower limit of 95%*CI*	The upper limit of 95%*CI*
**Model 7 (Inclusion of Eastern China residents only)**	**Extraversion (control group = High score group)**							
	**Low score group**	−0.013	0.0225	0.327	0.568	0.987	0.945	1.032
	**Agreeableness (control group = High score group)**							
	**Low score group**	−0.091	0.0245	13.777	**<0.001**	0.913	0.870	0.958
	**Conscientiousness (control group = High score group)**							
	**Low score group**	−0.092	0.0243	14.225	**<0.001**	0.912	0.870	0.957
	**Neuroticism (control group = High score group)**							
	**Low score group**	−0.018	0.0243	0.560	0.454	0.982	0.936	1.030
	**Openness (control group = High score group)**							
	**Low score group**	−0.042	0.0230	3.397	0.065	0.958	0.916	1.003
	**Health Literacy (control group = High score group)**							
	**Low score group**	−0.077	0.0255	9.105	**0.003**	0.926	0.881	0.973
	**EQ-VAS (control group = High score group)**							
	**Low score group**	−0.117	0.0239	24.018	**<0.001**	0.889	0.849	0.932
**Model 8(Inclusion of Central China residents only)**	**Extraversion (control group = High score group)**							
	**Low score group**	−0.051	0.0336	2.284	0.131	0.950	0.890	1.015
	**Agreeableness (control group = High score group)**							
	**Low score group**	−0.133	0.0348	14.517	**<0.001**	0.876	0.818	0.938
	**Conscientiousness (control group = High score group)**							
	**Low score group**	−0.074	0.0352	4.394	**0.036**	0.929	0.867	0.995
	**Neuroticism (control group = High score group)**							
	**Low score group**	−0.080	0.0354	5.057	**0.025**	0.923	0.862	0.990
	**Openness (control group = High score group)**							
	**Low score group**	−0.055	0.0341	2.616	0.106	0.946	0.885	1.012
	**Health Literacy (control group = High score group)**							
	**Low score group**	−0.008	0.0353	0.047	0.828	0.992	0.926	1.063
	**EQ-VAS (control group = High score group)**							
	**Low score group**	−0.096	0.0357	7.257	**0.007**	0.908	0.847	0.974
**Model 9 (Inclusion of Western China residents only)**	**Extraversion (control group = High score group)**							
	**Low score group**	−0.035	0.0317	1.222	0.269	0.966	0.907	1.027
	**Agreeableness (control group = High score group)**							
	**Low score group**	−0.151	0.0353	18.244	**<0.001**	0.860	0.802	0.922
	**Conscientiousness (control group = High score group)**							
	**Low score group**	−0.023	0.0337	0.478	0.489	0.977	0.915	1.044
	**Neuroticism (control group = High score group)**							
	**Low score group**	−0.061	0.0329	3.444	0.063	0.941	0.882	1.003
	**Openness (control group = High score group)**							
	**Low score group**	−0.087	0.0321	7.335	**0.007**	0.917	0.861	0.976
	**Health Literacy (control group = High score group)**							
	**Low score group**	0.002	0.0332	0.003	0.957	1.002	0.939	1.069
	**EQ-VAS (control group = High score group)**							
	**Low score group**	−0.034	0.0330	1.050	0.305	0.967	0.906	1.031

All models adjust factors gender, age, education level, the main way of medical expenses borne, place of residence, monthly income, marital Status, employment status, chronic diseases condition. The number in bold is *p*<0.05.

**TABLE 6:** Results of analysis of subgroups by region (dependent variable: likelihood of residents focusing on efficacy).

Models	Variables	β	SE	*Wald χ* ^*2*^	*p*	PRR	The lower limit of 95%*CI*	The upper limit of 95%*CI*
**Model 10 (Inclusion of Eastern China residents only)**	**Extraversion (control group = High score group)**							
	**Low score group**	−0.029	0.0242	1.412	0.235	0.972	0.927	1.019
	**Agreeableness (control group = High score group)**							
	**Low score group**	−0.059	0.0259	5.099	**0.024**	0.943	0.896	0.992
	**Conscientiousness (control group = High score group)**							
	**Low score group**	−0.138	0.0264	27.295	**<0.001**	0.871	0.827	0.917
	**Neuroticism (control group = High score group)**							
	**Low score group**	−0.037	0.0258	2.075	0.150	0.963	0.916	1.014
	**Openness (control group = High score group)**							
	**Low score group**	−0.048	0.0247	3.770	0.052	0.953	0.908	1.000
	**Health Literacy (control group = High score group)**							
	**Low score group**	−0.043	0.0267	2.543	0.111	0.958	0.909	1.010
	**EQ-VAS (control group = High score group)**							
	**Low score group**	−0.098	0.0255	14.764	**<0.001**	0.907	0.862	0.953
**Model 11 (Inclusion of Central China residents only)**	**Extraversion (control group = High score group)**							
	**Low score group**	0.013	0.0392	0.114	0.736	1.013	0.938	1.094
	**Agreeableness (control group = High score group)**							
	**Low score group**	−0.088	0.0402	4.766	**0.029**	0.916	0.846	0.991
	**Conscientiousness (control group = High score group)**							
	**Low score group**	−0.104	0.0409	6.468	**0.011**	0.901	0.832	0.976
	**Neuroticism (control group = High score group)**							
	**Low score group**	0.003	0.0423	0.005	0.946	1.003	0.923	1.090
	**Openness (control group = High score group)**							
	**Low score group**	−0.073	0.0387	3.535	0.060	0.930	0.862	1.003
	**Health Literacy (control group = High score group)**							
	**Low score group**	0.006	0.0395	0.025	0.874	1.006	0.931	1.087
	**EQ-VAS (control group = High score group)**							
	**Low score group**	−0.104	0.0406	6.517	**0.011**	0.902	0.833	0.976
**Model 12 (Inclusion of Western China residents only)**	**Extraversion (control group = High score group)**							
	**Low score group**	−0.009	0.0349	0.072	0.789	0.991	0.925	1.061
	**Agreeableness (control group = High score group)**							
	**Low score group**	−0.215	0.0398	29.093	**<0.001**	0.807	0.746	0.872
	**Conscientiousness (control group = High score group)**							
	**Low score group**	−0.121	0.0397	9.222	**0.002**	0.886	0.820	0.958
	**Neuroticism (control group = High score group)**							
	**Low score group**	0.016	0.0409	0.158	0.691	1.016	0.938	1.101
	**Openness (control group = High score group)**							
	**Low score group**	−0.032	0.0363	0.797	0.372	0.968	0.902	1.040
	**Health Literacy (control group = High score group)**							
	**Low score group**	−0.017	0.0378	0.205	0.651	0.983	0.913	1.059
	**EQ-VAS (control group = High score group)**							
	**Low score group**	−0.043	0.0372	1.332	0.248	0.958	0.891	1.030

All models adjust factors gender, age, education level, the main way of medical expenses borne, place of residence, monthly income, marital Status, employment status, chronic diseases condition. The number in bold is *p*<0.05.

In terms of whether to focus on safety, the influencing factors of personal character, health literacy and health status for eastern residents were agreeableness, conscientiousness, health literacy, and self-rated quality of life; for the central region, the relevant factors were agreeableness, conscientiousness, neuroticism, and self-rated quality of life; for the western region, the relevant factors were agreeableness, and openness.

In terms of whether to focus on efficacy, the influencing factors of personal character, health literacy and health status for eastern residents were agreeableness, conscientiousness, self-rated quality of life; for the central region, the relevant factors were agreeableness, conscientiousness and self-rated quality of life; and for the western region, the relevant factors were agreeableness, and conscientiousness.

## 4 Discussion

### 4.1 Current situation of self-medication in mainland China residents

Self-medication is becoming an increasingly common behavior, and people are becoming more independent in making decisions about their health management. Self-medication can help reduce the cost of producing, selling, and administering prescription drugs. However, people may suffer from misdiagnosis, prolonged or insufficient medication, neglect of drug interactions, and overdose due to their improper self-medication behaviors, which can delay their recovery process and even have serious, life-threatening consequences (Hughes et al., 2001).

Self-medication is a common practice around the world. The prevalence of self medication in Thailand reached 88.2% (Chautrakarn et al., 2021). The prevalence of self medication in the Indian population ranges from 8.3% to 93% (Rashid et al., 2020). 67.3% of people in Syria take drugs by themselves (Abdelwahed et al., 2022). In a study involving several European countries, self-medication rates among residents ranged from 33% in Turkey to 92% in the Czech Republic and 97% in Cyprus (Kamekis et al., 2018). This indicates that in many countries and regions, a large number of residents purchase and use OTC medicines by themselves as an important means of treating their diseases. Self-medication is also prevalent in China. The self-medication rate among the respondents in this study was 99.1%. The two most common types of OTC drugs purchased and used by the respondents were antipyretic and analgesics (5421 people, 58.6%) and vitamins/minerals (4851 people, 52.4%). Long et al. found that the self-medication rate of urban residents in China was 73.5%. A 2017 study by Chang J et al. showed that in China, 32.7% of people aged 45 and older used OTC for self-treatment within 4 weeks before the survey (Chang et al., 2017). Many studies have shown that self medication is related to sociodemographic characteristics and economic conditions (Subashini and Udayanga, 2020; Zeid et al., 2020).

### 4.2 Analysis of influencing factors of residents taking drug safety or efficacy as important considerations

The safety and efficacy of drugs are important considerations for most residents purchasing OTC drugs (Hanna and Hughes, 2011). A global study covering 51 countries reported similar findings that consumers value safety (“I know it is safe”) and effectiveness (“I know it works”) as the most important considerations while buying OTC drugs ^42^. This study showed that the number of respondents who purchased OTC medicines mainly considered drug safety as an important factor reached 5901, accounting for 63.7%, and the number of people who took drug efficacy as an important consideration reached 5492, accounting for 59.3%. An investigation of Australian consumers’ considerations for purchasing OTC drugs also showed that most consumers cite efficacy (1420/1627, 87%) and safety (1348/1625, 83%) as important considerations for purchasing OTC drugs (Bevan et al., 2019). In Japan, a developed region in Asia, information about self medication can be obtained from the Internet and government publicity. People with high quality of life can manage their symptoms according to their knowledge (Ohta et al., 2022). German research also shows that providing more OTC information for patients may improve patient safety (Eickhoff C,. et al., 2012). Self medication is associated with more severe disease grade and lower economic level of quality of life (Parvinroo et al., 2022).

We used log-binomial regression to analyze the associated factors of the dependent variables. In the log-binomial regression analysis of safety, gender, location, employment status, the main way of medical expenses borne, agreeableness, conscientiousness, neuroticism, openness, health literacy, and self-rated health status were significantly associated with the likelihood of drug safety being a consideration when purchasing OTC drugs.

Self-medication behaviors carry potential risks, so safety should be an important consideration when engaging in self-medication, and safety awareness should be an important part of how patients can manage their health. In this study, females were more likely to consider drug safety as an important consideration. This finding is in line with a previous study on drug safety awareness, in which males were more willing to accept risks, while females were more likely to seek information about drugs and place greater importance on safety (See et al., 2020).

Respondents who were retired were more likely to pay great attention to drug safety. Because retired people were elder and pays attention to safety, and has a certain level of knowledge and social experience, so that can make decisions on drug safety. Respondents those who mainly used ways including basic medical insurance for employees, commercial medical insurance, free medical treatment to cover their medical cost were more likely to consider drug safety as an important factor. People who participate in these types of insurance have a stronger risk awareness, and will pay more attention to safety when reimbursement funds are available.

Personality largely determines patients’ health behaviors, and when purchasing OTC drugs, personality may influence how much importance patients place on them. In this study, we found that people with high agreeableness, high conscientiousness, high neuroticism, and high openness were more likely to regard drug safety as an important consideration. High conscientiousness is a state of caution or vigilance that shows a desire to do something well (Vlieland et al., 2019). Therefore, people with high conscientiousness will pay more attention to the potential risks of OTC drugs to their health and are sensitive to safety. A higher level of neuroticism predicts anxiety due to worry-related problems (Beyer et al., 2015). This group is more worried about the adverse consequences when buying OTC drugs and thinks that drug safety is extremely important. People with high agreeableness and high openness will consider the advice of professionals more and make choices about their safety. Health beliefs are closely related to self-medication (Ting et al., 2018). People with high health literacy and self-assessed health levels have stronger health beliefs and safety awareness and are more likely to pay attention to drug safety when purchasing OTC drugs.

Another important factor in purchasing OTC drugs is the efficacy of the drug. In the log-binomial regression analysis of efficacy, we found that gender, location, employment status, the main way of medical expenses borne, chronic disease, agreeableness, conscientiousness, openness, and self-rated health status were all related to drug efficacy when respondents purchased OTC drugs. Many studies have shown that efficacy (usually determined by personal experience or advice from health professionals) is one of the factors most valued by consumers when considering safety (Hanna and Hughes, 2011). Compared with males, females cited drug efficacy as an important consideration when purchasing OTC medications (*p* < 0.05). Females visit pharmacies more frequently and may receive more information about drugs. A previous study in Sweden showed that females paid more attention to drug efficacy (Håkonsen et al., 2020). Compared to people without chronic diseases, people with chronic diseases pay more attention to drug efficacy due to the need for frequent medication, and they need cost-effective and effective medicines to treat diseases and relieve symptoms. Respondents who were unemployed were less likely to consider drug efficacy as an important factor. The knowledge level and social experience of unemployed are low, and their understanding of the practicality of drugs is insufficient (Chiappini et al., 2022). Respondents whose main way of medical expenses borne was Out-of-pocket Payment, were more likely to consider drug efficacy as an important factor, and this was because this group pays more attention to the cost-effectiveness (Lai et al., 2021).

### 4.3 Consideration of safety and efficacy in self-medication in eastern, central, and western regions

When residents self-medicate, the proportion of residents in the east, central and western regions who consider safety is 63.5%, 61.5%, and 66.8% respectively. The proportion of curative effect was 60.2%, 55.7%, and 61.4% respectively. The results of multifactor analysis showed that the residents in the west considered the safety of medication more than those in the east, while the residents in the middle considered the efficacy less than those in the east.

Stratified analysis of individual personality, health literacy, and the impact of self-assessment of health status on safety effectiveness in the eastern, central and western regions shows that in the eastern region, residents who are more agreeableness, more conscientiousness, and have higher health literacy, and who have higher self-rated quality of life pay more attention to safety. In the central region, residents with agreeableness, more conscientiousness, more neurotic and higher self-rated quality of life pay more attention to safety. In the western region, more agreeableness, and openness residents pay more attention to safety. In terms of curative effect, in the east, residents with more agreeableness, conscientious and self-rated quality of life pay more attention to drug effect. In the central region, residents with more agreeableness, conscientious and higher self-rated quality pay more attention to drug effect. For the western region, residents with more agreeableness and conscientious pay more attention to effect.

With the expansion of China’s regional economic development gap, the supply of medical and health services is unbalanced in the east, central and western regions, and the supply level of medical and health services in the eastern region is the highest (Cavaco et al., 2018). There is inequality in the distribution of economic and medical resources among different regions in China, and there are significant differences in the practice and guarantee of drug safety among regions. The study found that the drug safety practice of pharmacists in regions with high *per capita* GDP and rich medical resources is better than that in regions with low *per capita* GDP and relatively scarce medical resources (Tang L, 2012). Because the safety practice of drug use in the western region is lower than that in the central region, they are more likely to be unable to receive timely treatment due to the fear of adverse drug use events and when they experience adverse drug events such as anaphylactic shock. This is why western respondents tend to take drug safety as an important consideration compared with eastern respondents.

The research shows that in China, the score of openness in the western region is higher than that in other regions and provinces, and the openness of the central and western residents of the Big Five personality is more obvious (Xu L et al., 2022). The results of this study show that when self-medication, the openness characteristics of the Big Five personality of Chinese residents also appear in the western region, and the western residents with high openness are more concerned about the safety of self-medication. The agreeableness and conscientious personality traits significantly predict drug compliance (Abu EK L et al., 2022), suggesting that agreeableness and conscientious traits can significantly affect residents’ ability to make healthy choices. A sense of responsibility can enhance healthy behaviors, and a high sense of responsibility is related to beneficial self-care behaviors (Skinner et al., 2014). Therefore, both eastern and western regions show that the more pleasant and responsible they are, the more attention they pay to safety and effectiveness in self-medication.

People in the eastern region are changing their living habits and improving their health management ability. The overall health literacy in the central and western regions is not high. We should pay attention to strengthening health education and popularizing health knowledge. Therefore, residents with different levels of health literacy only show differences in the eastern region when considering the safety of self-medication behavior (Wang F et al., 2022). Both the multifactor analysis of safety and effectiveness and the subgroup analysis of the region showed that the patient compliance EQ-VAS was positively correlated with the health compliance medication behavior. Social determinants are closely related to health and play an important role in health-related quality of life. The eastern provinces of China are better than the central and western provinces in terms of population density, culture, economic status, and social infrastructure, affecting the significant differences in health-related quality of life between the eastern provinces and the central and western provinces. The EQ-VAS of residents in the east is higher, which significantly affects the consideration of the safety and effectiveness of self-medication.

High agreeableness, high conscientiousness, high openness, and high self-rated health status group were more likely to consider drug efficacy as an important factor. People with high conscientiousness pay more attention to whether OTC drugs would achieve a beneficial effect on the body. When purchasing OTC medicines, people with high agreeableness and high openness will listen more to the explanations of professionals and pay more attention to drugs when communicating with medical staff. People with high-level of self-assessed health status are in better health and therefore will pay more attention to their health and consider whether the drug can restore and maintain their health quickly and effectively when taking medication.

### 4.4 Advantages and limitations of the study

This study has several advantages. First, we obtained extensive and representative data using cross-sectional survey data across mainland China in 2021. In addition, this study combined the Big Five personality, health literacy, and health-related quality of life theories to analyze residents’ purchase behavior of OTC drugs. Theories such as the Big Five have made empirical contributions and expanded the value of new theoretical applications of self-medication health behaviors. At the same time, it makes targeted suggestions for the future practice of self-medication supervision and health education. It makes further practical contributions.

The study also has several limitations. First, the data were based exclusively on self-report questionnaires, which may be influenced by social expectations, self-report errors, and poor memory. Second, this study used a cross-sectional design, and the results were only used to explore the correlates of the dependent variables. Third, the participants in this study were all located in China, and the sample was homogeneous and cannot be generalized to other countries. We used the most recent data available to us (2021), however, the behavioral characteristics of residents and the important factors considered in OTC drug purchases are likely to change further in the ensuing years. Also, Due to the limited length of the paper, only two important properties of the drugs themself, namely, drug efficacy and safety, are discussed in this paper. Other considerations for purchasing OTC drugs will be further investigated in follow-up studies.

## 5 Relevance to clinical practice

Self-medication plays an important role in disease prevention, health promotion, and the treatment of mild illnesses. Consumers can obtain OTC drugs without a prescription, which allows them to access treatment more quickly and can reduce the burden on the healthcare system. However, there are certain potential risks associated with self-medication. For example, when residents take drugs by themselves, they are prone to overdose, underdose, and wrong medication.

For China, with a large population and great differences in health literacy, it is necessary to strengthen the management of OTC drugs and health education on public self-medication. We make the following recommendations for the health sector, pharmaceutical manufacturers and distributors, the media, medical personnel and the general public. The health sector needs to build a regulatory system, a partnership between doctors, patients and pharmacists, strengthen behavioral supervision and education medical staff. Drug manufacturers and distributors should recognize that drugs are a special commodity, and ensure that the drugs produced and sold are safe, effective, stable, and controllable in quality. Relevant media should publicize drug knowledge and common sense, and increase public opinion supervision on violations. Medical staff should improve their professional ability, strengthen training and professional experience and always play an important role in imparting knowledge to consumers. Pharmacy staff should provide more guidance on drug safety and efficacy to assist patients in choosing drugs. Residents should improve their health literacy, reserve drug-related knowledge and common sense, read the instruction of the drugs before using them, understand the indications and adverse reactions of commonly used OTC medications and take self-medication behaviors cautiously.

## 6 Conclusions

Self-medication is widespread among Chinese adults. The two most common OTC drugs that people buy and use on their own are antipyretic analgesics and vitamins/minerals. When Chinese adults buy OTC drugs, the safety and efficacy of the drug and the doctor’s recommendations are important considerations. The likelihood that people consider drug efficacy and safety as important considerations when purchasing OTC drugs is influenced by their demographic sociological characteristics, health literacy, self-assessed health status, and personality characteristics. The health sector should take measures to strengthen the management of OTC drugs, Pharmacy staff should provide more guidance on drug safety and efficacy to assist patients in choosing drugs. Improve residents’ drug-related health literacy, and enhance their attention to drug efficacy and safety, thereby reducing inappropriate self-medication behaviors and injuries and deaths caused by these inappropriate behaviors.

## Data Availability

The raw data supporting the conclusions of this article will be made available by the authors, without undue reservation.

## Abbreviations

OTC, Over-the-counter; HLS-SF, The health literacy scale-short form; BFI-10, The 10-item Big Five Inventory; FDA, The U.S. Food and Drug Administration; EQ-VAS, The EQ-5D visual analogue scale; RBMI, Resident Basic Medical Insurance
