# Supplementary material for: The self-medication behaviors of residents and the factors related to the consideration of drug efficacy and safety—A cross-sectional study in China
Source: Front Pharmacol. 2023 Feb 28;14:1072917. doi: 10.3389/fphar.2023.1072917 (PMC10011170; doi:10.3389/fphar.2023.1072917)
Supplement: Supplementary file 1 [file Table1.DOCX]

**Supplementary Table 1.** Chi-square test results of whether respondents consider drug safety as an important consideration when purchasing OTC drugs

|  |  | **whether respondents consider drug safety as an important consideration when purchasing OTC drugs** | | ***χ²（P）*** |
| --- | --- | --- | --- | --- |
|  | Number（Percentage） | No | Yes |  |
| **Gender** |  |  |  | 22.906（*P*<0.001） |
| Male | 4289（46.3%） | 1665（38.8%） | 2624（61.2%） |  |
| Female | 4967（53.7%） | 1690（34.0%） | 3277（66.0%） |  |
| **Age(years)** |  |  |  | 19.063（*P*＜0.001） |
| 19-35 | 4246（45.9%） | 1546（36.4%） | 2700（63.6%） |  |
| 36-59 | 3935（42.5%） | 1360（34.6%） | 2575（65.4%） |  |
| ≥60 | 1075（11.6%） | 449（41.8%） | 626（58.2%） |  |
| **Education level** |  |  |  | 7.579（*P*=0.056） |
| High/Secondary School and lower | 3685（39.8%） | 1379（37.4%） | 2306（62.6%） |  |
| Junior college | 1300（14.0%） | 452（34.8%） | 848（65.2%） |  |
| Undergraduate | 3654（39.5%） | 1283（35.1%） | 2371（64.9%） |  |
| Postgraduate degree | 617（6.7%） | 241（39.1%） | 376（60.9%） |  |
| **Location** |  |  |  | 14.025（*P*=0.001） |
| Eastern part of China | 4722（51.0%） | 1724（36.5%） | 2998（63.5%） |  |
| Central part of China | 2391（25.8%） | 920（38.5%） | 1471（61.5%） |  |
| Western part of China | 2143（23.2%） | 711（33.2%） | 1432（66.8%） |  |
| **Place of residence** |  |  |  | 0.000（*P*=0.996） |
| Urban | 2582（27.9%） | 936（36.3%） | 1646（63.7%） |  |
| Rural | 6674（72.1%） | 2419（36.2%） | 4255（63.8%） |  |
| **Marital Status** |  |  |  | 7.554（*P*=0.056） |
| Unmarried | 5765（62.3%） | 2071（35.9%） | 3694（64.1%） |  |
| Married | 3072（33.2%） | 1106（36.0%） | 1966（64.0%） |  |
| Divorced | 193（2.1%） | 80（41.5%） | 113（58.5%） |  |
| Widowed | 226（2.4%） | 98（43.4%） | 128（56.6%） |  |
| **Employment status** |  |  |  | 4.214（*P*=0.239） |
| Employed | 4129（44.6%） | 1467（35.5%） | 2662（64.5%） |  |
| Student | 2144（23.2%） | 766（35.7%） | 1378（64.3%） |  |
| Unemployed | 2174（23.5%） | 827（38.0%） | 1347（62.0%） |  |
| Retired | 809（8.7%） | 295（36.5%） | 514（63.5%） |  |
| **The main way of medical expenses borne** |  |  |  | 14.587（*P*=0.001） |
| Out-of-pocket payments | 1840（19.9%） | 695（37.8%） | 1145（62.2%） |  |
| Resident Basic Medical Insurance (RBMI) | 4472（48.3%） | 1675（37.5%） | 2797（62.5%） |  |
| Others | 2944（31.8%） | 985（33.5%） | 1959（66.5%） |  |
| **Whether chronic disease is diagnosed at present** |  |  |  | 8.570（*P*=0.003） |
| No | 7357（79.5%） | 2612（35.5%） | 4745（64.5%） |  |
| Yes | 1899（20.5%） | 743（39.1%） | 1156（60.9%） |  |
| **Monthly income (RMB ¥)** |  |  |  | 0.029（*P*=0.985） |
| 0-4500（0$-666$） | 4735（51.2%） | 1714（36.2%） | 3021（63.8%） |  |
| 4501-9000（666.148$-1332$） | 3146（34.0%） | 1144（36.4%） | 2002（63.6%） |  |
| ＞9000（1332$） | 1375（14.8%） | 497（36.1%） | 878（63.9%） |  |
| **Extraversion** |  |  |  | 24.904（*P*<0.001） |
| High score group | 3349（36.2%） | 1103（32.9%） | 2246（67.1%） |  |
| Low score group | 5907（63.8%） | 2252（38.1%） | 3655（61.9%） |  |
| **Agreeableness** |  |  |  | 117.924（*P*<0.001） |
| High score group | 5182（56.0%） | 1629（31.4%） | 3553（68.6%） |  |
| Low score group | 4074（44.0%） | 1726（42.4%） | 2348（57.6%） |  |
| **Conscientiousness** |  |  |  | 75.807（*P*<0.001） |
| High score group | 4757（51.4%） | 1523（32.0%） | 3234（68.0%） |  |
| Low score group | 4499（48.6%） | 1832（40.7%） | 2667（59.3%） |  |
| **Neuroticism** |  |  |  | 6.110（*P*=0.013） |
| High score group | 2257（24.4%） | 769（34.1%） | 1488（65.9%） |  |
| Low score group | 6999（75.6%） | 2586（36.9%） | 4413（63.1%） |  |
| **Openness** |  |  |  | 60.598（*P*<0.001） |
| High score group | 3565（38.5%） | 1117（31.3%） | 2448（68.7%） |  |
| Low score group | 5691（61.5%） | 2238（39.3%） | 3453（60.7%） |  |
| **Health literacy** |  |  |  | 21.909（*P*＜0.001） |
| High score group | 5942（64.2%） | 2050（34.5%） | 3892（65.5%） |  |
| Low score group | 3314（35.8%） | 1305（39.4%） | 2009（60.6%） |  |
| **EQ-5D-VAS** |  |  |  | 62.666（*P*<0.001） |
| High score group | 5460（59.0%） | 1799（32.9%） | 3661（67.1%） |  |
| Low score group | 3796（41.0%） | 1556（41.0%） | 2240（59.0%） |  |

**Supplementary Table 2.** Chi-square test results of whether respondents consider drug efficacy as an important consideration when purchasing OTC drugs

|  |  | **whether respondents consider drug efficacy as an important consideration when purchasing OTC drugs** | | ***χ²（P）*** |
| --- | --- | --- | --- | --- |
|  | Number（Percentage） | No | Yes |  |
| **Gender** |  |  |  | 16.545（*P*<0.001） |
| Male | 4289（46.3%） | 1840（42.9%） | 2449（57.1%） |  |
| Female | 4967（53.7%） | 1924（38.7%） | 3043（61.3%） |  |
| **Age(years)** |  |  |  | 6.379（*P*=0.041） |
| 19-35 | 4246（45.9%） | 1786（42.1%） | 2460（57.9%） |  |
| 36-59 | 3935（42.5%） | 1556（39.5%） | 2379（60.5%） |  |
| ≥60 | 1075（11.6%） | 422（39.3%） | 653（60.7%） |  |
| **Education level** |  |  |  | 1.776（*P*=0.620） |
| High/Secondary School and lower | 3685（39.8%） | 1511（41.0%） | 2174（59.0%） |  |
| Junior college | 1300（14.0%） | 514（39.5%） | 786（60.5%） |  |
| Undergraduate | 3654（39.5%） | 1477（40.4%） | 2177（59.6%） |  |
| Postgraduate degree | 617（6.7%） | 262（42.5%） | 355（57.5%） |  |
| **Location** |  |  |  | 18.319 （*P*<0.001） |
| Eastern part of China | 4722（51.0%） | 1877（39.8%） | 2845（60.2%） |  |
| Central part of China | 2391（25.8%） | 1059（44.3%） | 1332（55.7%） |  |
| Western part of China | 2143（23.2%） | 828（38.6%） | 1315（61.4%） |  |
| **Place of residence** |  |  |  | 0.222 （*P*=0.638） |
| Urban | 2582（27.9%） | 1040（40.3%） | 1542（59.7%） |  |
| Rural | 6674（72.1%） | 2724（40.8%） | 3950（59.2%） |  |
| **Marital Status** |  |  |  | 7.986 （*P*=0.046） |
| Unmarried | 5765（62.3%） | 2286（39.7%） | 3479（60.3%） |  |
| Married | 3072（33.2%） | 1304（42.4%） | 1768（57.6%） |  |
| Divorced | 193（2.1%） | 86（44.6%） | 107（55.4%） |  |
| Widowed | 226（2.4%） | 88（38.9%） | 138（61.1%） |  |
| **Employment status** |  |  |  | 14.961 （*P*=0.002） |
| Employed | 4129（44.6%） | 1626（39.4%） | 2503（60.6%） |  |
| Student | 2144（23.2%） | 903（42.1%） | 1241（57.9%） |  |
| Unemployed | 2174（23.5%） | 937（43.1%） | 1237（56.9%） |  |
| Retired | 809（8.7%） | 298（36.8%） | 511（63.2%） |  |
| **The main way of medical expenses borne** |  |  |  | 4.548 （*P*=0.103） |
| Out-of-pocket payments | 1840（19.9%） | 755（41.0%） | 1085（59.0%） |  |
| Resident Basic Medical Insurance (RBMI) | 4472（48.3%） | 1858（41.5%） | 2614（58.5%） |  |
| Others | 2944（31.8%） | 1151（39.1%） | 1793（60.9%） |  |
| **Whether chronic disease is diagnosed at present** |  |  |  | 4.899（*P*=0.027） |
| No | 7357（79.5%） | 3034（41.2%） | 4323（58.8%） |  |
| Yes | 1899（20.5%） | 730（38.4%） | 1169（61.6%） |  |
| **Monthly income (RMB ¥)** |  |  |  | 1.981（*P*=0.371） |
| 0-4500（0$-666$） | 4735（51.2%） | 1904（40.2%） | 2831（59.8%） |  |
| 4501-9000（666.148$-1332$） | 3146（34.0%） | 1278（40.6%） | 1868（59.4%） |  |
| ＞9000（1332$） | 1375（14.8%） | 582（42.3%） | 793（57.7%） |  |
| **Extraversion** |  |  |  | 14.305（*P*<0.001） |
| High score group | 3349（36.2%） | 1276（38.1%） | 2073（61.9%） |  |
| Low score group | 5907（63.8%） | 2488（42.1%） | 3419（57.9%） |  |
| **Agreeableness** |  |  |  | 99.740（*P*<0.001） |
| High score group | 5182（56.0%） | 1873（36.1%） | 3309（63.9%） |  |
| Low score group | 4074（44.0%） | 1891（46.4%） | 2183（53.6%） |  |
| **Conscientiousness** |  |  |  | 116.05（*P*<0.001） |
| High score group | 4757（51.4%） | 1680（35.3%） | 3077（64.7%） |  |
| Low score group | 4499（48.6%） | 2084（46.3%） | 2415（53.7%） |  |
| **Neuroticism** |  |  |  | 0.860（*P*=0.354） |
| High score group | 2257（24.4%） | 899（39.8%） | 1358（60.2%） |  |
| Low score group | 6999（75.6%） | 2865（40.9%） | 4134（59.1%） |  |
| **Openness** |  |  |  | 35.865（*P*<0.001） |
| High score group | 3565（38.5%） | 1312（36.8%） | 2253（63.2%） |  |
| Low score group | 5691（61.5%） | 2452（43.1%） | 3239（56.9%） |  |
| **Health literacy** |  |  |  | 5.136（*P*=0.023） |
| High score group | 5942（64.2%） | 2365（39.8%） | 3577（60.2%） |  |
| Low score group | 3314（35.8%） | 1399（42.2%） | 1915（57.8%） |  |
| **EQ-5D-VAS** |  |  |  | 41.277（*P*<0.001） |
| High score group | 5460（59.0%） | 2071（37.9%） | 3389（62.1%） |  |
| Low score group | 3796（41.0%） | 1693（44.6%） | 2103（55.4%） |  |

**Supplementary Table 3.** Log-binomial regression results of male residents with drug safety as the dependent variable

| **Variables** | **β** | **SE** | ***Wald χ²*** | ***P*** | **PRR** | **The lower limit of 95%*CI*** | **The upper limit of 95%*CI*** |
| --- | --- | --- | --- | --- | --- | --- | --- |
| **Age（control group =19-35）** |  |  |  |  |  |  |  |
| **36-59** | 0.030 | 0.0355 | 0.728 | 0.394 | 1.031 | 0.961 | 1.105 |
| **60 or above** | -0.028 | 0.0610 | 0.206 | 0.650 | 0.973 | 0.863 | 1.096 |
| **Educational level（control group =High/Secondary School and lower）** |  |  |  |  |  |  |  |
| **Junior college** | 0.037 | 0.0353 | 1.126 | 0.289 | 1.038 | 0.969 | 1.112 |
| **Undergraduate** | -0.019 | 0.0329 | 0.348 | 0.555 | 0.981 | 0.920 | 1.046 |
| **Postgraduate** | -0.100 | 0.0571 | 3.050 | 0.081 | 0.905 | 0.809 | 1.012 |
| **Location（control group =Eastern part of China）** |  |  |  |  |  |  |  |
| **Central part of China** | 0.014 | 0.0295 | 0.229 | 0.632 | 1.014 | 0.957 | 1.075 |
| **Western part of China** | 0.098 | 0.0278 | 12.372 | ＜**0.001** | 1.103 | 1.044 | 1.164 |
| **Place of residence（control group =Rural）** |  |  |  |  |  |  |  |
| **Urban** | -0.031 | 0.0284 | 1.231 | 0.267 | 0.969 | 0.917 | 1.024 |
| **Marital Status（control group =Unmarried）** |  |  |  |  |  |  |  |
| **Married** | -0.024 | 0.0416 | 0.324 | 0.569 | 0.977 | 0.900 | 1.060 |
| **Divorced** | -0.200 | 0.1124 | 3.173 | 0.075 | 0.819 | 0.657 | 1.020 |
| **Widowed** | 0.067 | 0.0911 | 0.546 | 0.460 | 1.070 | 0.895 | 1.279 |
| **Employment status（control group =Employed）** |  |  |  |  |  |  |  |
| **Student** | -0.024 | 0.0545 | 0.194 | 0.659 | 0.976 | 0.877 | 1.086 |
| **Unemployed** | 0.032 | 0.0363 | 0.755 | 0.385 | 1.032 | 0.961 | 1.108 |
| **Retired** | 0.069 | 0.0544 | 1.617 | 0.203 | 1.072 | 0.963 | 1.192 |
| **The main way of medical expenses borne（control group =Out-of-pocket Payment）** |  |  |  |  |  |  |  |
| **Resident Basic Medical Insurance (RBMI)** | -0.027 | 0.0343 | 0.627 | 0.429 | 0.973 | 0.910 | 1.041 |
| **Others（Basic medical insurance for employees, Commercial medical insurance, Free medical treatment）** | 0.062 | 0.0389 | 2.499 | 0.114 | 1.063 | 0.985 | 1.148 |
| **Chronic diseases condition（control group =No chronic diseases）** |  |  |  |  |  |  |  |
| **Suffer from chronic diseases** | -0.035 | 0.0314 | 1.256 | 0.262 | 0.965 | 0.908 | 1.027 |
| **Monthly income (RMB )（control group =0-4500（0$-666$））** |  |  |  |  |  |  |  |
| **4501-9000（666.148$-1332$）** | 0.013 | 0.0274 | 0.209 | 0.648 | 1.013 | 0.960 | 1.068 |
| **＞9000（1332$）** | -0.005 | .0373 | 0.021 | 0.885 | 0.995 | 0.925 | 1.070 |
| **Extraversion（control group =High score group）** |  |  |  |  |  |  |  |
| **Low score group** | -0.038 | 0.0254 | 2.225 | 0.136 | 0.963 | 0.916 | 1.012 |
| **Agreeableness（control group =High score group）** |  |  |  |  |  |  |  |
| **Low score group** | -0.137 | 0.0274 | 24.995 | **＜0.001** | 0.872 | 0.826 | 0.920 |
| **Conscientiousness（control group =High score group）** |  |  |  |  |  |  |  |
| **Low score group** | -0.082 | 0.0275 | 8.861 | 0.003 | 0.922 | 0.873 | 0.972 |
| **Neuroticism（control group =High score group）** |  |  |  |  |  |  |  |
| **Low score group** | -0.022 | 0.0292 | 0.564 | 0.453 | 0.978 | 0.924 | 1.036 |
| **Openness（control group =High score group）** |  |  |  |  |  |  |  |
| **Low score group** | -0.064 | 0.0259 | 6.144 | 0.013 | 0.938 | 0.891 | 0.987 |
| **Health Literacy（control group =High score group）** |  |  |  |  |  |  |  |
| **Low score group** | -0.038 | 0.0266 | 2.038 | 0.153 | 0.963 | 0.914 | 1.014 |
| **EQ-VAS（control group =High score group）** |  |  |  |  |  |  |  |
| **Low score group** | -0.108 | 0.0267 | 16.251 | **＜0.001** | 0.898 | 0.852 | 0.946 |

**Supplementary Table 4.** Log-binomial regression results of female residents with drug safety as the dependent variable

| **Variables** | **β** | **SE** | ***Wald χ²*** | ***P*** | **PRR** | **The lower limit of 95%*CI*** | **The upper limit of 95%*CI*** |
| --- | --- | --- | --- | --- | --- | --- | --- |
| **Age（control group =19-35）** |  |  |  |  |  |  |  |
| **36-59** | 0.041 | 0.0313 | 1.720 | 0.190 | 1.042 | 0.980 | 1.108 |
| **60 or above** | -0.119 | 0.0564 | 4.432 | 0.035 | 0.888 | 0.795 | 0.992 |
| **Educational level（control group =High/Secondary School and lower）** |  |  |  |  |  |  |  |
| **Junior college** | 0.003 | 0.0338 | 0.006 | 0.936 | 1.003 | 0.938 | 1.071 |
| **Undergraduate** | -0.005 | 0.0304 | 0.029 | 0.865 | 0.995 | 0.937 | 1.056 |
| **Postgraduate** | -0.030 | 0.0492 | 0.375 | 0.540 | 0.970 | 0.881 | 1.068 |
| **Location（control group =Eastern part of China）** |  |  |  |  |  |  |  |
| **Central part of China** | -0.058 | 0.0258 | 5.067 | 0.024 | 0.944 | 0.897 | 0.993 |
| **Western part of China** | 0.001 | 0.0248 | 0.001 | 0.980 | 1.001 | 0.953 | 1.051 |
| **Place of residence（control group =Rural）** |  |  |  |  |  |  |  |
| **Urban** | -0.023 | 0.0240 | 0.910 | 0.340 | 0.977 | 0.932 | 1.024 |
| **Marital Status（control group =Unmarried）** |  |  |  |  |  |  |  |
| **Married** | 0.052 | 0.0383 | 1.816 | 0.178 | 1.053 | 0.977 | 1.135 |
| **Divorced** | -0.054 | 0.0681 | 0.622 | 0.430 | 0.948 | 0.829 | 1.083 |
| **Widowed** | -0.096 | 0.0812 | 1.407 | 0.236 | 0.908 | 0.775 | 1.065 |
| **Employment status（control group =Employed）** |  |  |  |  |  |  |  |
| **Student** | 0.010 | 0.0385 | 0.066 | 0.797 | 1.010 | 0.937 | 1.089 |
| **Unemployed** | 0.035 | 0.0314 | 1.272 | 0.259 | 1.036 | 0.974 | 1.102 |
| **Retired** | 0.088 | 0.0443 | 3.957 | 0.047 | 1.092 | 1.001 | 1.191 |
| **The main way of medical expenses borne（control group =Out-of-pocket Payment）** |  |  |  |  |  |  |  |
| **Resident Basic Medical Insurance (RBMI)** | 0.002 | 0.0270 | 0.005 | 0.942 | 1.002 | 0.950 | 1.056 |
| **Others（Basic medical insurance for employees, Commercial medical insurance, Free medical treatment）** | 0.038 | 0.0335 | 1.270 | 0.260 | 1.038 | 0.973 | 1.109 |
| **Chronic diseases condition（control group =No chronic diseases）** |  |  |  |  |  |  |  |
| **Suffer from chronic diseases** | 0.010 | 0.0299 | 0.104 | 0.747 | 1.010 | 0.952 | 1.071 |
| **Monthly income (RMB )（control group =0-4500（0$-666$））** |  |  |  |  |  |  |  |
| **4501-9000（666.148$-1332$）** | -0.038 | 0.0235 | 2.604 | 0.107 | 0.963 | 0.919 | 1.008 |
| **＞9000（1332$）** | 0.017 | 0.0311 | 0.316 | 0.574 | 1.018 | 0.957 | 1.082 |
| **Extraversion（control group =High score group）** |  |  |  |  |  |  |  |
| **Low score group** | -0.020 | 0.0208 | 0.881 | 0.348 | 0.981 | 0.942 | 1.021 |
| **Agreeableness（control group =High score group）** |  |  |  |  |  |  |  |
| **Low score group** | -0.089 | 0.0227 | 15.222 | **＜0.001** | 0.915 | 0.875 | 0.957 |
| **Conscientiousness（control group =High score group）** |  |  |  |  |  |  |  |
| **Low score group** | -0.066 | 0.0222 | 8.899 | 0.003 | 0.936 | 0.896 | 0.978 |
| **Neuroticism（control group =High score group）** |  |  |  |  |  |  |  |
| **Low score group** | -0.052 | 0.0213 | 6.027 | 0.014 | 0.949 | 0.910 | 0.990 |
| **Openness（control group =High score group）** |  |  |  |  |  |  |  |
| **Low score group** | -0.050 | 0.0213 | 5.460 | 0.019 | 0.951 | 0.913 | 0.992 |
| **Health Literacy（control group =High score group）** |  |  |  |  |  |  |  |
| **Low score group** | -0.045 | 0.0233 | 3.746 | 0.053 | 0.956 | 0.913 | 1.001 |
| **EQ-VAS（control group =High score group）** |  |  |  |  |  |  |  |
| **Low score group** | -0.077 | 0.0219 | 12.449 | **＜0.001** | 0.926 | 0.887 | 0.966 |

**Supplementary Table 5.** Log-binomial regression results of residents aged 35 and younger with drug safety as the dependent variable

| **Variables** | **β** | **SE** | ***Wald χ²*** | ***P*** | **PRR** | **The lower limit of 95%*CI*** | **The upper limit of 95%*CI*** |
| --- | --- | --- | --- | --- | --- | --- | --- |
| **Gender（control group =Male）** |  |  |  |  |  |  |  |
| **Female** | 0.075 | 0.0241 | 9.761 | 0.002 | 1.078 | 1.028 | 1.130 |
| **Educational level（control group =High/Secondary School and lower）** |  |  |  |  |  |  |  |
| **Junior college** | 0.038 | 0.0443 | 0.723 | 0.395 | 1.038 | 0.952 | 1.132 |
| **Undergraduate** | -0.005 | 0.0362 | 0.022 | .883 | 0.995 | 0.926 | 1.068 |
| **Postgraduate** | -0.043 | 0.0511 | 0.713 | 0.398 | 0.958 | 0.866 | 1.059 |
| **Location（control group =Eastern part of China）** |  |  |  |  |  |  |  |
| **Central part of China** | -0.017 | 0.0288 | 0.352 | 0.553 | 0.983 | 0.929 | 1.040 |
| **Western part of China** | 0.041 | 0.0273 | 2.202 | 0.138 | 1.041 | 0.987 | 1.099 |
| **Place of residence（control group =Rural）** |  |  |  |  |  |  |  |
| **Urban** | -0.028 | 0.0263 | 1.099 | 0.295 | 0.973 | 0.924 | 1.024 |
| **Marital Status（control group =Unmarried）** |  |  |  |  |  |  |  |
| **Married** | 0.026 | 0.0322 | 0.641 | 0.423 | 1.026 | 0.963 | 1.093 |
| **Divorced** | -0.272 | 0.1797 | 2.290 | 0.130 | 0.762 | 0.536 | 1.084 |
| **Widowed** | -0.773 | 0.8670 | 0.794 | 0.373 | 0.462 | 0.084 | 2.526 |
| **Employment status（control group =Employed）** |  |  |  |  |  |  |  |
| **Student** | -0.001 | 0.0335 | ＜0.001 | 0.983 | 0.999 | 0.936 | 1.067 |
| **Unemployed** | -0.007 | 0.0439 | 0.029 | 0.866 | 0.993 | 0.911 | 1.082 |
| **The main way of medical expenses borne（control group =Out-of-pocket Payment）** |  |  |  |  |  |  |  |
| **Resident Basic Medical Insurance (RBMI)** | 0.019 | 0.0277 | 0.454 | 0.500 | 1.019 | 0.965 | 1.076 |
| **Others（Basic medical insurance for employees, Commercial medical insurance, Free medical treatment）** | 0.053 | 0.0366 | 2.091 | 0.148 | 1.054 | 0.981 | 1.133 |
| **Chronic diseases condition（control group =No chronic diseases）** |  |  |  |  |  |  |  |
| **Suffer from chronic diseases** | -0.150 | 0.0623 | 5.824 | 0.016 | 0.860 | 0.762 | 0.972 |
| **Monthly income (RMB )（control group =0-4500（0$-666$））** |  |  |  |  |  |  |  |
| **4501-9000（666.148$-1332$）** | -0.016 | 0.0267 | 0.344 | 0.557 | 0.984 | 0.934 | 1.037 |
| **＞9000（1332$）** | -0.049 | 0.0271 | 3.269 | 0.071 | 0.952 | 0.903 | 1.004 |
| **Extraversion（control group =High score group）** |  |  |  |  |  |  |  |
| **Low score group** | -0.058 | 0.0242 | 5.763 | 0.016 | 0.944 | 0.900 | 0.989 |
| **Agreeableness（control group =High score group）** |  |  |  |  |  |  |  |
| **Low score group** | -0.101 | 0.0260 | 15.063 | ＜0.001 | 0.904 | 0.859 | 0.951 |
| **Conscientiousness（control group =High score group）** |  |  |  |  |  |  |  |
| **Low score group** | -0.017 | 0.0243 | 0.488 | 0.485 | 0.983 | 0.937 | 1.031 |
| **Neuroticism（control group =High score group）** |  |  |  |  |  |  |  |
| **Low score group** | -0.031 | 0.0251 | 1.521 | 0.217 | 0.969 | 0.923 | 1.018 |
| **Openness（control group =High score group）** |  |  |  |  |  |  |  |
| **Low score group** | -0.091 | 0.0251 | 13.009 | ＜0.001 | 0.913 | 0.869 | 0.959 |
| **Health Literacy（control group =High score group）** |  |  |  |  |  |  |  |
| **Low score group** | -0.010 | 0.0269 | 0.130 | 0.718 | 0.990 | 0.940 | 1.044 |
| **EQ-VAS（control group =High score group）** |  |  |  |  |  |  |  |
| **Low score group** | -0.065 | 0.0252 | 6.718 | 0.010 | 0.937 | 0.891 | 0.984 |
|  |  |  |  |  |  |  |  |

**Supplementary Table 6.** Log-binomial regression results of residents over the age of 35 with drug safety as the dependent variable

| **Variables** | **β** | **SE** | ***Wald χ²*** | ***P*** | **PRR** | **The lower limit of 95%*CI*** | **The upper limit of 95%*CI*** |
| --- | --- | --- | --- | --- | --- | --- | --- |
| **Gender（control group =Male）** |  |  |  |  |  |  |  |
| **Female** | 0.030 | 0.0208 | 2.072 | 0.150 | 1.030 | 0.989 | 1.073 |
| **Educational level（control group =High/Secondary School and lower）** |  |  |  |  |  |  |  |
| **Junior college** | 0.013 | 0.0305 | 0.169 | 0.681 | 1.013 | 0.954 | 1.075 |
| **Undergraduate** | -0.023 | 0.0305 | 0.550 | 0.458 | 0.978 | 0.921 | 1.038 |
| **Postgraduate** | -0.079 | 0.0612 | 1.662 | 0.197 | 0.924 | 0.820 | 1.042 |
| **Location（control group =Eastern part of China）** |  |  |  |  |  |  |  |
| **Central part of China** | -0.035 | 0.0260 | 1.832 | 0.176 | 0.965 | 0.918 | 1.016 |
| **Western part of China** | 0.043 | 0.0248 | 3.015 | 0.082 | 1.044 | 0.994 | 1.096 |
| **Place of residence（control group =Rural）** |  |  |  |  |  |  |  |
| **Urban** | -0.024 | 0.0261 | 0.826 | 0.364 | 0.977 | 0.928 | 1.028 |
| **Marital Status（control group =Unmarried）** |  |  |  |  |  |  |  |
| **Married** | -0.074 | 0.0706 | 1.102 | 0.294 | 0.929 | 0.809 | 1.066 |
| **Divorced** | -0.072 | 0.0613 | 1.364 | 0.243 | 0.931 | 0.826 | 1.050 |
| **Widowed** | -0.092 | 0.0593 | 2.387 | 0.122 | 0.912 | 0.812 | 1.025 |
| **Employment status（control group =Employed）** |  |  |  |  |  |  |  |
| **Unemployed** | 0.033 | 0.0290 | 1.298 | 0.254 | 1.034 | 0.977 | 1.094 |
| **Retired** | 0.036 | 0.0312 | 1.303 | 0.254 | 1.036 | 0.975 | 1.102 |
| **The main way of medical expenses borne（control group =Out-of-pocket Payment）** |  |  |  |  |  |  |  |
| **Resident Basic Medical Insurance (RBMI)** | -0.052 | 0.0328 | 2.553 | 0.110 | 0.949 | 0.890 | 1.012 |
| **Others（Basic medical insurance for employees, Commercial medical insurance, Free medical treatment）** | 0.032 | 0.0364 | 0.763 | 0.382 | 1.032 | 0.961 | 1.109 |
| **Chronic diseases condition（control group =No chronic diseases）** |  |  |  |  |  |  |  |
| **Suffer from chronic diseases** | -0.002 | 0.0234 | 0.007 | 0.933 | 0.998 | 0.953 | 1.045 |
| **Monthly income (RMB )（control group =0-4500（0$-666$））** |  |  |  |  |  |  |  |
| **4501-9000（666.148$-1332$）** | -0.008 | 0.0243 | 0.115 | 0.734 | 0.992 | 0.946 | 1.040 |
| **＞9000（1332$）** | 0.043 | 0.0336 | 1.663 | 0.197 | 1.044 | 0.978 | 1.115 |
| **Extraversion（control group =High score group）** |  |  |  |  |  |  |  |
| **Low score group** | ＜0.001 | .0216 | ＜0.001 | .982 | 1.000 | 0.958 | 1.043 |
| **Agreeableness（control group =High score group）** |  |  |  |  |  |  |  |
| **Low score group** | -0.101 | 0.0240 | 17.566 | ＜0.001 | 0.904 | 0.863 | 0.948 |
| **Conscientiousness（control group =High score group）** |  |  |  |  |  |  |  |
| **Low score group** | -0.120 | 0.0254 | 22.451 | ＜0.001 | 0.887 | 0.844 | 0.932 |
| **Neuroticism（control group =High score group）** |  |  |  |  |  |  |  |
| **Low score group** | -0.051 | 0.0234 | 4.816 | 0.028 | 0.950 | 0.907 | 0.995 |
| **Openness（control group =High score group）** |  |  |  |  |  |  |  |
| **Low score group** | -0.039 | 0.0220 | 3.168 | 0.075 | 0.962 | 0.921 | 1.004 |
| **Health Literacy（control group =High score group）** |  |  |  |  |  |  |  |
| **Low score group** | -0.067 | 0.0230 | 8.449 | 0.004 | 0.935 | 0.894 | 0.978 |
| **EQ-VAS（control group =High score group）** |  |  |  |  |  |  |  |
| **Low score group** | -0.111 | 0.0228 | 23.640 | ＜0.001 | 0.895 | 0.856 | 0.936 |

**Supplementary Table 7.** Log-binomial regression results of rural residents with drug safety as the dependent variable

| **Variables** | **β** | **SE** | ***Wald χ²*** | ***P*** | **PRR** | **The lower limit of 95%*CI*** | **The upper limit of 95%*CI*** |
| --- | --- | --- | --- | --- | --- | --- | --- |
| **Gender（control group =Male）** |  |  |  |  |  |  |  |
| **Female** | 0.041 | 0.0301 | 1.820 | 0.177 | 1.041 | 0.982 | 1.105 |
| **Age（control group =19-35）** |  |  |  |  |  |  |  |
| **36-59** | 0.067 | 0.0488 | 1.911 | 0.167 | 1.070 | 0.972 | 1.177 |
| **60 or above** | -0.122 | 0.0697 | 3.038 | 0.081 | 0.886 | 0.772 | 1.015 |
| **Educational level（control group =High/Secondary School and lower）** |  |  |  |  |  |  |  |
| **Junior college** | 0.016 | 0.0586 | 0.079 | 0.779 | 1.017 | 0.906 | 1.140 |
| **Undergraduate** | 0.008 | 0.0469 | 0.027 | 0.869 | 1.008 | 0.919 | 1.105 |
| **Postgraduate** | -0.069 | 0.1045 | 0.441 | 0.507 | 0.933 | 0.760 | 1.145 |
| **Location（control group =Eastern part of China）** |  |  |  |  |  |  |  |
| **Central part of China** | 0.016 | 0.0356 | 0.201 | 0.654 | 1.016 | 0.948 | 1.089 |
| **Western part of China** | 0.014 | 0.0351 | 0.163 | 0.687 | 1.014 | 0.947 | 1.086 |
| **Marital Status（control group =Unmarried）** |  |  |  |  |  |  |  |
| **Married** | 0.052 | 0.0570 | 0.830 | 0.362 | 1.053 | 0.942 | 1.178 |
| **Divorced** | -0.141 | 0.1438 | 0.961 | 0.327 | 0.869 | 0.655 | 1.151 |
| **Widowed** | -0.008 | 0.0887 | 0.008 | 0.930 | 0.992 | 0.834 | 1.181 |
| **Employment status（control group =Employed）** |  |  |  |  |  |  |  |
| **Student** | 0.067 | 0.0631 | 1.116 | 0.291 | 1.069 | 0.945 | 1.210 |
| **Unemployed** | 0.090 | 0.0428 | 4.390 | 0.036 | 1.094 | 1.006 | 1.190 |
| **Retired** | 0.176 | 0.0706 | 6.202 | 0.013 | 1.192 | 1.038 | 1.369 |
| **The main way of medical expenses borne（control group =Out-of-pocket Payment）** |  |  |  |  |  |  |  |
| **Resident Basic Medical Insurance (RBMI)** | 0.035 | 0.0366 | 0.942 | 0.332 | 1.036 | 0.964 | 1.113 |
| **Others（Basic medical insurance for employees, Commercial medical insurance, Free medical treatment）** | 0.083 | 0.0536 | 2.422 | 0.120 | 1.087 | 0.979 | 1.207 |
| **Chronic diseases condition（control group =No chronic diseases）** |  |  |  |  |  |  |  |
| **Suffer from chronic diseases** | -0.029 | 0.0414 | 0.501 | 0.479 | 0.971 | 0.895 | 1.053 |
| **Monthly income (RMB )（control group =0-4500（0$-666$））** |  |  |  |  |  |  |  |
| **4501-9000（666.148$-1332$）** | -0.021 | 0.0363 | 0.339 | 0.560 | 0.979 | 0.912 | 1.051 |
| **＞9000（1332$）** | 0.027 | 0.0582 | 0.216 | 0.642 | 1.027 | 0.917 | 1.152 |
| **Extraversion（control group =High score group）** |  |  |  |  |  |  |  |
| **Low score group** | -0.103 | 0.0304 | 11.445 | 0.001 | 0.902 | 0.850 | 0.958 |
| **Agreeableness（control group =High score group）** |  |  |  |  |  |  |  |
| **Low score group** | -.0100 | 0.0336 | 8.881 | 0.003 | 0.905 | 0.847 | 0.966 |
| **Conscientiousness（control group =High score group）** |  |  |  |  |  |  |  |
| **Low score group** | -0.052 | 0.0333 | 2.430 | 0.119 | 0.949 | 0.889 | 1.013 |
| **Neuroticism（control group =High score group）** |  |  |  |  |  |  |  |
| **Low score group** | -0.020 | 0.0328 | 0.384 | 0.536 | 0.980 | 0.919 | 1.045 |
| **Openness（control group =High score group）** |  |  |  |  |  |  |  |
| **Low score group** | -0.076 | 0.0315 | 5.758 | 0.016 | 0.927 | 0.872 | 0.986 |
| **Health Literacy（control group =High score group）** |  |  |  |  |  |  |  |
| **Low score group** | -0.009 | 0.0312 | 0.075 | 0.784 | 0.991 | 0.933 | 1.054 |
| **EQ-VAS（control group =High score group）** |  |  |  |  |  |  |  |
| **Low score group** | -0.058 | 0.0308 | 3.599 | 0.058 | 0.943 | 0.888 | 1.002 |

**Supplementary Table 8.** Log-binomial regression results of urban residents with drug safety as the dependent variable

| **Variables** | **β** | **SE** | ***Wald χ²*** | ***P*** | **PRR** | **The lower limit of 95%*CI*** | **The upper limit of 95%*CI*** |
| --- | --- | --- | --- | --- | --- | --- | --- |
| **Gender（control group =Male）** |  |  |  |  |  |  |  |
| **Female** | 0.053 | 0.0184 | 8.262 | 0.004 | 1.054 | 1.017 | 1.093 |
| **Age（control group =19-35）** |  |  |  |  |  |  |  |
| **36-59** | 0.020 | 0.0265 | 0.588 | 0.443 | 1.021 | 0.969 | 1.075 |
| **60 or above** | -0.035 | 0.0531 | 0.428 | 0.513 | 0.966 | 0.870 | 1.072 |
| **Educational level（control group =High/Secondary School and lower）** |  |  |  |  |  |  |  |
| **Junior college** | 0.019 | 0.0275 | 0.492 | 0.483 | 1.019 | 0.966 | 1.076 |
| **Undergraduate** | -0.023 | 0.0253 | 0.814 | 0.367 | 0.977 | 0.930 | 1.027 |
| **Postgraduate** | -0.070 | 0.0403 | 3.060 | 0.080 | 0.932 | 0.861 | 1.009 |
| **Location（control group =Eastern part of China）** |  |  |  |  |  |  |  |
| **Central part of China** | -0.047 | 0.0229 | 4.255 | 0.039 | 0.954 | 0.912 | 0.998 |
| **Western part of China** | 0.054 | 0.0214 | 6.418 | 0.011 | 1.056 | 1.012 | 1.101 |
| **Marital Status（control group =Unmarried）** |  |  |  |  |  |  |  |
| **Married** | 0.006 | 0.0316 | 0.040 | 0.841 | 1.006 | 0.946 | 1.071 |
| **Divorced** | -0.085 | 0.0647 | 1.732 | 0.188 | 0.918 | 0.809 | 1.043 |
| **Widowed** | -0.064 | 0.0852 | 0.562 | 0.454 | 0.938 | 0.794 | 1.109 |
| **Employment status（control group =Employed）** |  |  |  |  |  |  |  |
| **Student** | 0.012 | 0.0338 | 0.116 | 0.733 | 1.012 | 0.947 | 1.081 |
| **Unemployed** | -0.002 | 0.0299 | 0.007 | 0.935 | 0.998 | 0.941 | 1.058 |
| **Retired** | 0.043 | 0.0422 | 1.036 | 0.309 | 1.044 | 0.961 | 1.134 |
| **The main way of medical expenses borne（control group =Out-of-pocket Payment）** |  |  |  |  |  |  |  |
| **Resident Basic Medical Insurance (RBMI)** | -0.024 | 0.0262 | 0.821 | 0.365 | 0.977 | 0.928 | 1.028 |
| **Others（Basic medical insurance for employees, Commercial medical insurance, Free medical treatment）** | 0.032 | 0.0293 | 1.207 | 0.272 | 1.033 | 0.975 | 1.094 |
| **Chronic diseases condition（control group =No chronic diseases）** |  |  |  |  |  |  |  |
| **Suffer from chronic diseases** | -0.010 | 0.0254 | 0.149 | 0.699 | 0.990 | 0.942 | 1.041 |
| **Monthly income (RMB )（control group =0-4500（0$-666$））** |  |  |  |  |  |  |  |
| **4501-9000（666.148$-1332$）** | -0.017 | 0.0204 | 0.665 | 0.415 | 0.983 | 0.945 | 1.024 |
| **＞9000（1332$）** | -0.002 | 0.0263 | 0.005 | 0.946 | 0.998 | 0.948 | 1.051 |
| **Extraversion（control group =High score group）** |  |  |  |  |  |  |  |
| **Low score group** | 0.003 | 0.0189 | 0.021 | 0.886 | 1.003 | 0.966 | 1.041 |
| **Agreeableness（control group =High score group）** |  |  |  |  |  |  |  |
| **Low score group** | -0.111 | 0.0207 | 28.553 | ＜0.001 | 0.895 | 0.860 | 0.932 |
| **Conscientiousness（control group =High score group）** |  |  |  |  |  |  |  |
| **Low score group** | -0.076 | 0.0202 | 14.104 | ＜0.001 | 0.927 | 0.891 | 0.964 |
| **Neuroticism（control group =High score group）** |  |  |  |  |  |  |  |
| **Low score group** | -0.048 | 0.0200 | 5.851 | 0.016 | 0.953 | 0.916 | 0.991 |
| **Openness（control group =High score group）** |  |  |  |  |  |  |  |
| **Low score group** | -0.056 | 0.0193 | 8.581 | 0.003 | 0.945 | 0.910 | 0.981 |
| **Health Literacy（control group =High score group）** |  |  |  |  |  |  |  |
| **Low score group** | -0.057 | 0.0213 | 7.274 | 0.007 | 0.944 | 0.906 | 0.984 |
| **EQ-VAS（control group =High score group）** |  |  |  |  |  |  |  |
| **Low score group** | -0.107 | 0.0204 | 27.447 | ＜0.001 | 0.899 | 0.864 | 0.935 |

**Supplementary Table 9.** Log-binomial regression results of male residents with drug efficacy as the dependent variable

| **Variables** | **β** | **SE** | ***Wald χ²*** | ***P*** | **PRR** | **The lower limit of 95%*CI*** | **The upper limit of 95%*CI*** |
| --- | --- | --- | --- | --- | --- | --- | --- |
| **Age（control group =19-35）** |  |  |  |  |  |  |  |
| **36-59** | 0.030 | 0.0394 | 0.581 | 0.446 | 1.030 | 0.954 | 1.113 |
| **60 or above** | 0.083 | 0.0663 | 1.582 | 0.208 | 1.087 | 0.955 | 1.238 |
| **Educational level（control group =High/Secondary School and lower）** |  |  |  |  |  |  |  |
| **Junior college** | 0.052 | 0.0413 | 1.604 | 0.205 | 1.054 | 0.972 | 1.143 |
| **Undergraduate** | 0.007 | 0.0367 | 0.035 | 0.853 | 1.007 | 0.937 | 1.082 |
| **Postgraduate** | -0.015 | 0.0613 | 0.062 | 0.804 | 0.985 | 0.873 | 1.111 |
| **Location（control group =Eastern part of China）** |  |  |  |  |  |  |  |
| **Central part of China** | -0.097 | 0.0326 | 8.821 | 0.003 | 0.908 | 0.851 | 0.968 |
| **Western part of China** | 0.030 | 0.0309 | 0.910 | 0.340 | 1.030 | 0.969 | 1.094 |
| **Place of residence（control group =Rural）** |  |  |  |  |  |  |  |
| **Urban** | -0.063 | 0.0308 | 4.177 | 0.041 | 0.939 | 0.884 | 0.997 |
| **Marital Status（control group =Unmarried）** |  |  |  |  |  |  |  |
| **Married** | -0.060 | 0.0480 | 1.545 | 0.214 | 0.942 | 0.858 | 1.035 |
| **Divorced** | -0.087 | 0.1075 | 0.650 | 0.420 | 0.917 | 0.743 | 1.132 |
| **Widowed** | -0.064 | 0.1043 | 0.373 | 0.541 | 0.938 | 0.765 | 1.151 |
| **Employment status（control group =Employed）** |  |  |  |  |  |  |  |
| **Student** | -0.068 | 0.0426 | 2.559 | 0.110 | 0.934 | 0.859 | 1.015 |
| **Unemployed** | -0.068 | 0.0426 | 2.559 | 0.110 | 0.934 | 0.859 | 1.015 |
| **Retired** | 0.010 | 0.0643 | 0.022 | 0.882 | 1.010 | 0.890 | 1.145 |
| **The main way of medical expenses borne（control group =Out-of-pocket Payment）** |  |  |  |  |  |  |  |
| **Resident Basic Medical Insurance (RBMI)** | -0.049 | 0.0357 | 1.874 | 0.171 | 0.952 | 0.888 | 1.021 |
| **Others（Basic medical insurance for employees, Commercial medical insurance, Free medical treatment）** | -0.071 | 0.0430 | 2.715 | 0.099 | 0.932 | 0.856 | 1.014 |
| **Chronic diseases condition（control group =No chronic diseases）** |  |  |  |  |  |  |  |
| **Suffer from chronic diseases** | 0.057 | 0.0329 | 2.951 | 0.086 | 1.058 | 0.992 | 1.129 |
| **Monthly income (RMB )（control group =0-4500（0$-666$））** |  |  |  |  |  |  |  |
| **4501-9000（666.148$-1332$）** | -0.029 | 0.0300 | 0.958 | 0.328 | 0.971 | 0.916 | 1.030 |
| **＞9000（1332$）** | -0.090 | 0.0407 | 4.858 | 0.028 | 0.914 | 0.844 | 0.990 |
| **Extraversion（control group =High score group）** |  |  |  |  |  |  |  |
| **Low score group** | ..013 | ..0278 | ..222 | ..638 | 1.013 | ..959 | 1.070 |
| **Agreeableness（control group =High score group）** |  |  |  |  |  |  |  |
| **Low score group** | -0.123 | 0.0296 | 17.301 | **＜0.001** | 0.884 | 0.834 | 0.937 |
| **Conscientiousness（control group =High score group）** |  |  |  |  |  |  |  |
| **Low score group** | -0.159 | 0.0299 | 28.089 | **＜0.001** | 0.853 | 0.805 | 0.905 |
| **Neuroticism（control group =High score group）** |  |  |  |  |  |  |  |
| **Low score group** | 0.039 | 0.0333 | 1.376 | 0.241 | 1.040 | 0.974 | 1.110 |
| **Openness（control group =High score group）** |  |  |  |  |  |  |  |
| **Low score group** | -0.062 | 0.0281 | 4.953 | 0.026 | 0.939 | 0.889 | 0.993 |
| **Health Literacy（control group =High score group）** |  |  |  |  |  |  |  |
| **Low score group** | -0.072 | 0.0299 | 5.808 | 0.016 | 0.930 | 0.878 | 0.987 |
| **EQ-VAS（control group =High score group）** |  |  |  |  |  |  |  |
| **Low score group** | -0.053 | 0.0289 | 3.359 | 0.067 | 0.948 | 0.896 | 1.004 |

**Supplementary Table 10.** Log-binomial regression results of female residents with drug efficacy as the dependent variable

| **Variables** | **β** | **SE** | ***Wald χ²*** | ***P*** | **PRR** | **The lower limit of 95%*CI*** | **The upper limit of 95%*CI*** |
| --- | --- | --- | --- | --- | --- | --- | --- |
| **Age（control group =19-35）** |  |  |  |  |  |  |  |
| **36-59** | -0.002 | 0.0336 | 0.003 | 0.954 | 0.998 | 0.934 | 1.066 |
| **60 or above** | -0.033 | 0.0596 | 0.297 | 0.586 | 0.968 | 0.861 | 1.088 |
| **Educational level（control group =High/Secondary School and lower）** |  |  |  |  |  |  |  |
| **Junior college** | 0.015 | 0.0380 | 0.149 | 0.699 | 1.015 | 0.942 | 1.093 |
| **Undergraduate** | 0.014 | 0.0344 | 0.177 | 0.674 | 1.015 | 0.948 | 1.085 |
| **Postgraduate** | -0.008 | 0.0548 | 0.019 | 0.889 | 0.992 | 0.891 | 1.105 |
| **Location（control group =Eastern part of China）** |  |  |  |  |  |  |  |
| **Central part of China** | -0.084 | 0.0286 | 8.591 | 0.003 | 0.919 | 0.869 | 0.973 |
| **Western part of China** | -0.005 | 0.0275 | 0.029 | 0.866 | 0.995 | 0.943 | 1.050 |
| **Place of residence（control group =Rural）** |  |  |  |  |  |  |  |
| **Urban** | **＜0.001** | 0.0270 | **＜0.001** | 0.993 | 1.000 | 0.949 | 1.055 |
| **Marital Status（control group =Unmarried）** |  |  |  |  |  |  |  |
| **Married** | 0.014 | 0.0417 | 0.116 | 0.733 | 1.014 | 0.935 | 1.101 |
| **Divorced** | -0.082 | 0.0793 | 1.080 | 0.299 | 0.921 | 0.788 | 1.076 |
| **Widowed** | 0.068 | 0.0698 | 0.945 | 0.331 | 1.070 | 0.933 | 1.227 |
| **Employment status（control group =Employed）** |  |  |  |  |  |  |  |
| **Student** | -0.019 | 0.0441 | 0.185 | 0.667 | 0.981 | 0.900 | 1.070 |
| **Unemployed** | -0.057 | 0.0356 | 2.515 | 0.113 | 0.945 | 0.881 | 1.013 |
| **Retired** | 0.014 | 0.0485 | 0.084 | 0.772 | 1.014 | 0.922 | 1.115 |
| **The main way of medical expenses borne（control group =Out-of-pocket Payment）** |  |  |  |  |  |  |  |
| **Resident Basic Medical Insurance (RBMI)** | -0.053 | 0.0299 | 3.182 | 0.074 | 0.948 | 0.894 | 1.005 |
| **Others（Basic medical insurance for employees, Commercial medical insurance, Free medical treatment）** | -0.033 | 0.0368 | 0.792 | 0.374 | 0.968 | 0.900 | 1.040 |
| **Chronic diseases condition（control group =No chronic diseases）** |  |  |  |  |  |  |  |
| **Suffer from chronic diseases** | 0.079 | 0.0321 | 6.097 | 0.014 | 1.082 | 1.016 | 1.153 |
| **Monthly income (RMB )（control group =0-4500（0$-666$））** |  |  |  |  |  |  |  |
| **4501-9000（666.148$-1332$）** | -0.014 | 0.0257 | 0.295 | 0.587 | 0.986 | 0.938 | 1.037 |
| **＞9000（1332$）** | -0.004 | 0.0358 | 0.011 | 0.916 | 0.996 | 0.929 | 1.069 |
| **Extraversion（control group =High score group）** |  |  |  |  |  |  |  |
| **Low score group** | -0.021 | 0.0232 | 0.811 | 0.368 | 0.979 | 0.936 | 1.025 |
| **Agreeableness（control group =High score group）** |  |  |  |  |  |  |  |
| **Low score group** | -0.083 | 0.0251 | 11.019 | 0.001 | 0.920 | 0.876 | 0.966 |
| **Conscientiousness（control group =High score group）** |  |  |  |  |  |  |  |
| **Low score group** | -.108 | .0256 | 17.843 | **＜0.001** | .898 | .854 | .944 |
| **Neuroticism（control group =High score group）** |  |  |  |  |  |  |  |
| **Low score group** | -0.035 | 0.0242 | 2.079 | 0.149 | 0.966 | 0.921 | 1.013 |
| **Openness（control group =High score group）** |  |  |  |  |  |  |  |
| **Low score group** | -0.042 | 0.0238 | 3.134 | 0.077 | 0.959 | 0.915 | 1.005 |
| **Health Literacy（control group =High score group）** |  |  |  |  |  |  |  |
| **Low score group** | 0.014 | 0.0249 | 0.309 | 0.578 | 1.014 | 0.966 | 1.065 |
| **EQ-VAS（control group =High score group）** |  |  |  |  |  |  |  |
| **Low score group** | -0.111 | 0.0248 | 19.983 | **＜0.001** | 0.895 | 0.853 | 0.940 |

**Supplementary Table 11.** Log-binomial regression results of residents aged 35 and younger with drug efficacy as the dependent variable

| **Variables** | **β** | **SE** | ***Wald χ²*** | ***P*** | **PRR** | **The lower limit of 95%*CI*** | **The upper limit of 95%*CI*** |
| --- | --- | --- | --- | --- | --- | --- | --- |
| **Gender（control group =Male）** |  |  |  |  |  |  |  |
| **Female** | 0.105 | 0.0274 | 14.668 | **＜0.001** | 1.110 | 1.052 | 1.172 |
| **Educational level（control group =High/Secondary School and lower）** |  |  |  |  |  |  |  |
| **Junior college** | 0.024 | 0.0508 | 0.219 | 0.640 | 1.024 | 0.927 | 1.131 |
| **Undergraduate** | 0.022 | 0.0419 | 0.271 | 0.603 | 1.022 | 0.941 | 1.110 |
| **Postgraduate** | 0.033 | 0.0565 | 0.347 | 0.556 | 1.034 | 0.925 | 1.155 |
| **Location（control group =Eastern part of China）** |  |  |  |  |  |  |  |
| **Central part of China** | -0.044 | 0.0329 | 1.786 | 0.181 | 0.957 | 0.897 | 1.021 |
| **Western part of China** | 0.048 | 0.0309 | 2.394 | 0.122 | 1.049 | 0.987 | 1.115 |
| **Place of residence（control group =Rural）** |  |  |  |  |  |  |  |
| **Urban** | -0.039 | 0.0297 | 1.723 | 0.189 | 0.962 | 0.907 | 1.019 |
| **Marital Status（control group =Unmarried）** |  |  |  |  |  |  |  |
| **Married** | 0.033 | 0.0360 | 0.828 | 0.363 | 1.033 | 0.963 | 1.109 |
| **Divorced** | -0.389 | 0.2136 | 3.311 | 0.069 | 0.678 | 0.446 | 1.030 |
| **Widowed** | 0.123 | 0.5022 | 0.060 | 0.806 | 1.131 | 0.423 | 3.026 |
| **Employment status（control group =Employed）** |  |  |  |  |  |  |  |
| **Student** | -0.065 | 0.0389 | 2.832 | 0.092 | 0.937 | 0.868 | 1.011 |
| **Unemployed** | -0.058 | 0.0495 | 1.349 | 0.245 | 0.944 | 0.857 | 1.040 |
| **The main way of medical expenses borne（control group =Out-of-pocket Payment）** |  |  |  |  |  |  |  |
| **Resident Basic Medical Insurance (RBMI)** | -.096 | 0.0302 | 10.104 | 0.001 | 0.908 | 0.856 | 0.964 |
| **Others（Basic medical insurance for employees, Commercial medical insurance, Free medical treatment）** | -.0105 | 0.0420 | 6.218 | 0.013 | 0.901 | 0.829 | 0.978 |
| **Chronic diseases condition（control group =No chronic diseases）** |  |  |  |  |  |  |  |
| **Suffer from chronic diseases** | 0.089 | 0.0531 | 2.807 | 0.094 | 1.093 | 0.985 | 1.213 |
| **Monthly income (RMB )（control group =0-4500（0$-666$））** |  |  |  |  |  |  |  |
| **4501-9000（666.148$-1332$）** | -0.011 | 0.0301 | 0.145 | 0.703 | 0.989 | 0.932 | 1.049 |
| **＞9000（1332$）** | -0.033 | 0.0402 | 0.675 | 0.411 | 0.967 | 0.894 | 1.047 |
| **Extraversion（control group =High score group）** |  |  |  |  |  |  |  |
| **Low score group** | -0.036 | 0.0273 | 1.716 | 0.190 | 0.965 | 0.915 | 1.018 |
| **Agreeableness（control group =High score group）** |  |  |  |  |  |  |  |
| **Low score group** | -0.097 | 0.0284 | 11.737 | 0.001 | 0.907 | 0.858 | 0.959 |
| **Conscientiousness（control group =High score group）** |  |  |  |  |  |  |  |
| **Low score group** | -0.100 | 0.0276 | 13.103 | **＜0.001** | 0.905 | 0.857 | 0.955 |
| **Neuroticism（control group =High score group）** |  |  |  |  |  |  |  |
| **Low score group** | -0.031 | 0.0290 | 1.152 | 0.283 | 0.969 | 0.916 | 1.026 |
| **Openness（control group =High score group）** |  |  |  |  |  |  |  |
| **Low score group** | -0.074 | 0.0281 | 6.962 | 0.008 | 0.929 | 0.879 | 0.981 |
| **Health Literacy（control group =High score group）** |  |  |  |  |  |  |  |
| **Low score group** | 0.009 | 0.0299 | 0.088 | 0.766 | 1.009 | 0.951 | 1.070 |
| **EQ-VAS（control group =High score group）** |  |  |  |  |  |  |  |
| **Low score group** | -0.048 | 0.0284 | 2.826 | 0.093 | 0.953 | 0.902 | 1.008 |
|  |  |  |  |  |  |  |  |

**Supplementary Table 12.** Log-binomial regression results of residents over the age of 35with drug efficacy as the dependent variable

| **Variables** | **β** | **SE** | ***Wald χ²*** | ***P*** | **PRR** | **The lower limit of 95%*CI*** | **The upper limit of 95%*CI*** |
| --- | --- | --- | --- | --- | --- | --- | --- |
| **Gender（control group =Male）** |  |  |  |  |  |  |  |
| **Female** | 0.011 | 0.0224 | 0.252 | 0.616 | 1.011 | 0.968 | 1.057 |
| **Educational level（control group =High/Secondary School and lower）** |  |  |  |  |  |  |  |
| **Junior college** | 0.041 | 0.0336 | 1.494 | 0.222 | 1.042 | 0.976 | 1.113 |
| **Undergraduate** | 0.008 | 0.0327 | 0.053 | 0.818 | 1.008 | 0.945 | 1.074 |
| **Postgraduate** | -0.056 | 0.0682 | 0.676 | 0.411 | 0.945 | 0.827 | 1.081 |
| **Location（control group =Eastern part of China）** |  |  |  |  |  |  |  |
| **Central part of China** | -0.123 | 0.0284 | 18.755 | **＜0.001** | 0.884 | 0.836 | 0.935 |
| **Western part of China** | -0.022 | 0.0273 | 0.648 | 0.421 | 0.978 | 0.927 | 1.032 |
| **Place of residence（control group =Rural）** |  |  |  |  |  |  |  |
| **Urban** | -0.018 | 0.0279 | 0.411 | 0.521 | 0.982 | 0.930 | 1.037 |
| **Marital Status（control group =Unmarried）** |  |  |  |  |  |  |  |
| **Married** | -0.287 | 0.0969 | 8.751 | 0.003 | 0.751 | 0.621 | 0.908 |
| **Divorced** | -0.037 | 0.0648 | 0.334 | 0.563 | 0.963 | 0.848 | 1.094 |
| **Widowed** | 0.023 | 0.0556 | 0.171 | 0.680 | 1.023 | 0.918 | 1.141 |
| **Employment status（control group =Employed）** |  |  |  |  |  |  |  |
| **Unemployed** | -0.043 | 0.0321 | 1.777 | 0.182 | 0.958 | 0.900 | 1.020 |
| **Retired** | 0.033 | 0.0326 | 1.003 | 0.317 | 1.033 | 0.969 | 1.101 |
| **The main way of medical expenses borne（control group =Out-of-pocket Payment）** |  |  |  |  |  |  |  |
| **Resident Basic Medical Insurance (RBMI)** | -0.002 | 0.0365 | 0.003 | 0.954 | 0.998 | 0.929 | 1.072 |
| **Others（Basic medical insurance for employees, Commercial medical insurance, Free medical treatment）** | -0.004 | 0.0411 | 0.008 | 0.928 | 0.996 | 0.919 | 1.080 |
| **Chronic diseases condition（control group =No chronic diseases）** |  |  |  |  |  |  |  |
| **Suffer from chronic diseases** | 0.069 | 0.0247 | 7.704 | 0.006 | 1.071 | 1.020 | 1.124 |
| **Monthly income (RMB )（control group =0-4500（0$-666$））** |  |  |  |  |  |  |  |
| **4501-9000（666.148$-1332$）** | -0.031 | 0.0256 | 1.499 | 0.221 | 0.969 | 0.922 | 1.019 |
| **＞9000（1332$）** | -0.046 | 0.0369 | 1.541 | 0.215 | 0.955 | 0.889 | 1.027 |
| **Extraversion（control group =High score group）** |  |  |  |  |  |  |  |
| **Low score group** | 0.004 | 0.0235 | 0.023 | 0.881 | 1.004 | 0.958 | 1.051 |
| **Agreeableness（control group =High score group）** |  |  |  |  |  |  |  |
| **Low score group** | -0.097 | 0.0263 | 13.726 | **＜0.001** | 0.907 | 0.862 | 0.955 |
| **Conscientiousness（control group =High score group）** |  |  |  |  |  |  |  |
| **Low score group** | -0.153 | 0.0281 | 29.557 | **＜0.001** | .858 | 0.812 | 0.907 |
| **Neuroticism（control group =High score group）** |  |  |  |  |  |  |  |
| **Low score group** | 0.017 | 0.0266 | 0.408 | 0.523 | 1.017 | 0.965 | 1.072 |
| **Openness（control group =High score group）** |  |  |  |  |  |  |  |
| **Low score group** | -0.025 | 0.0240 | 1.046 | 0.306 | 0.976 | 0.931 | 1.023 |
| **Health Literacy（control group =High score group）** |  |  |  |  |  |  |  |
| **Low score group** | -0.044 | 0.0245 | 3.196 | 0.074 | 0.957 | 0.912 | 1.004 |
| **EQ-VAS（control group =High score group）** |  |  |  |  |  |  |  |
| **Low score group** | -0.118 | 0.0246 | 23.040 | **＜0.001** | 0.889 | 0.847 | 0.932 |

**Supplementary Table 13.** Log-binomial regression results of rural residents with drug efficacy as the dependent variable

| **Variables** | **β** | **SE** | ***Wald χ²*** | ***P*** | **PRR** | **The lower limit of 95%*CI*** | **The upper limit of 95%*CI*** |
| --- | --- | --- | --- | --- | --- | --- | --- |
| **Gender（control group =Male）** |  |  |  |  |  |  |  |
| **Female** | 0.002 | 0.0318 | 0.003 | 0.959 | 1.002 | 0.941 | 1.066 |
| **Age（control group =19-35）** |  |  |  |  |  |  |  |
| **36-59** | 0.011 | 0.0531 | 0.044 | 0.833 | 1.011 | 0.911 | 1.122 |
| **60 or above** | 0.005 | 0.0740 | 0.004 | 0.948 | 1.005 | 0.869 | 1.162 |
| **Educational level（control group =High/Secondary School and lower）** |  |  |  |  |  |  |  |
| **Junior college** | 0.062 | 0.0627 | 0.963 | 0.326 | 1.063 | 0.940 | 1.203 |
| **Undergraduate** | 0.066 | 0.0549 | 1.462 | 0.227 | 1.069 | 0.960 | 1.190 |
| **Postgraduate** | 0.101 | 0.1043 | 0.932 | 0.334 | 1.106 | 0.901 | 1.357 |
| **Location（control group =Eastern part of China）** |  |  |  |  |  |  |  |
| **Central part of China** | -0.056 | 0.0415 | 1.847 | 0.174 | 0.945 | 0.871 | 1.025 |
| **Western part of China** | 0.036 | 0.0371 | 0.926 | 0.336 | 1.036 | 0.964 | 1.115 |
| **Marital Status（control group =Unmarried）** |  |  |  |  |  |  |  |
| **Married** | -0.043 | 0.0669 | 0.405 | 0.525 | 0.958 | 0.841 | 1.093 |
| **Divorced** | -0.255 | 0.1629 | 2.448 | 0.118 | 0.775 | 0.563 | 1.067 |
| **Widowed** | -0.028 | 0.0842 | 0.108 | 0.742 | 0.973 | 0.825 | 1.147 |
| **Employment status（control group =Employed）** |  |  |  |  |  |  |  |
| **Student** | -0.024 | 0.0720 | 0.115 | 0.734 | 0.976 | 0.847 | 1.124 |
| **Unemployed** | -0.093 | 0.0481 | 3.753 | 0.053 | 0.911 | 0.829 | 1.001 |
| **Retired** | 0.024 | 0.0780 | 0.092 | 0.762 | 1.024 | 0.879 | 1.193 |
| **The main way of medical expenses borne（control group =Out-of-pocket Payment）** |  |  |  |  |  |  |  |
| **Resident Basic Medical Insurance (RBMI)** | 0.017 | 0.0396 | 0.181 | 0.671 | 1.017 | 0.941 | 1.099 |
| **Others（Basic medical insurance for employees, Commercial medical insurance, Free medical treatment）** | -0.074 | 0.0628 | 1.399 | 0.237 | 0.928 | 0.821 | 1.050 |
| **Chronic diseases condition（control group =No chronic diseases）** |  |  |  |  |  |  |  |
| **Suffer from chronic diseases** | 0.068 | 0.0456 | 2.252 | 0.133 | 1.071 | 0.979 | 1.171 |
| **Monthly income (RMB )（control group =0-4500（0$-666$））** |  |  |  |  |  |  |  |
| **4501-9000（666.148$-1332$）** | -0.039 | 0.0401 | 0.955 | 0.329 | 0.962 | 0.889 | 1.040 |
| **＞9000（1332$）** | -0.015 | 0.0680 | 0.046 | 0.830 | 0.986 | 0.862 | 1.126 |
| **Extraversion（control group =High score group）** |  |  |  |  |  |  |  |
| **Low score group** | 0.036 | 0.0344 | 1.114 | 0.291 | 1.037 | 0.969 | 1.109 |
| **Agreeableness（control group =High score group）** |  |  |  |  |  |  |  |
| **Low score group** | -0.153 | 0.0370 | 17.177 | **＜0.001** | 0.858 | 0.798 | 0.922 |
| **Conscientiousness（control group =High score group）** |  |  |  |  |  |  |  |
| **Low score group** | -0.107 | 0.0366 | 8.558 | 0.003 | 0.898 | 0.836 | 0.965 |
| **Neuroticism（control group =High score group）** |  |  |  |  |  |  |  |
| **Low score group** | 0.018 | 0.0374 | 0.236 | 0.627 | 1.018 | 0.946 | 1.096 |
| **Openness（control group =High score group）** |  |  |  |  |  |  |  |
| **Low score group** | -0.071 | 0.0349 | 4.175 | 0.041 | 0.931 | 0.870 | 0.997 |
| **Health Literacy（control group =High score group）** |  |  |  |  |  |  |  |
| **Low score group** | 0.070 | 0.0352 | 3.943 | 0.047 | 1.072 | 1.001 | 1.149 |
| **EQ-VAS（control group =High score group）** |  |  |  |  |  |  |  |
| **Low score group** | -0.094 | 0.0349 | 7.289 | 0.007 | 0.910 | 0.850 | 0.975 |

**Supplementary Table 14.** Log-binomial regression results of urban residents with drug efficacy as the dependent variable

| **Variables** | **β** | **SE** | ***Wald χ²*** | ***P*** | **PRR** | **The lower limit of 95%*CI*** | **The upper limit of 95%*CI*** |
| --- | --- | --- | --- | --- | --- | --- | --- |
| **Gender（control group =Male）** |  |  |  |  |  |  |  |
| **Female** | 0.073 | 0.0204 | 12.980 | **＜0.001** | 1.076 | 1.034 | 1.120 |
| **Age（control group =19-35）** |  |  |  |  |  |  |  |
| **36-59** | 0.015 | 0.0291 | 0.261 | 0.610 | 1.015 | 0.959 | 1.075 |
| **60 or above** | 0.033 | 0.0557 | 0.353 | 0.553 | 1.034 | 0.927 | 1.153 |
| **Educational level（control group =High/Secondary School and lower）** |  |  |  |  |  |  |  |
| **Junior college** | 0.027 | 0.0314 | 0.730 | 0.393 | 1.027 | 0.966 | 1.092 |
| **Undergraduate** | 0.011 | 0.0283 | 0.153 | 0.696 | 1.011 | 0.957 | 1.069 |
| **Postgraduate** | -0.023 | 0.0445 | 0.257 | 0.612 | 0.978 | 0.896 | 1.067 |
| **Location（control group =Eastern part of China）** |  |  |  |  |  |  |  |
| **Central part of China** | -0.099 | 0.0251 | 15.503 | **＜0.001** | 0.906 | 0.862 | 0.952 |
| **Western part of China** | -0.003 | 0.0244 | 0.020 | 0.887 | 0.997 | 0.950 | 1.045 |
| **Marital Status（control group =Unmarried）** |  |  |  |  |  |  |  |
| **Married** | -0.013 | 0.0358 | 0.126 | 0.722 | 0.987 | 0.921 | 1.059 |
| **Divorced** | -0.057 | 0.0681 | 0.689 | 0.407 | 0.945 | 0.827 | 1.080 |
| **Widowed** | 0.045 | 0.0776 | 0.342 | 0.559 | 1.046 | 0.899 | 1.218 |
| **Employment status（control group =Employed）** |  |  |  |  |  |  |  |
| **Student** | -0.030 | 0.0397 | 0.574 | 0.449 | 0.970 | 0.898 | 1.049 |
| **Unemployed** | -0.054 | 0.0338 | 2.549 | 0.110 | 0.947 | 0.887 | 1.012 |
| **Retired** | 0.010 | 0.0450 | 0.050 | 0.823 | 1.010 | 0.925 | 1.103 |
| **The main way of medical expenses borne（control group =Out-of-pocket Payment）** |  |  |  |  |  |  |  |
| **Resident Basic Medical Insurance (RBMI)** | -0.086 | 0.0282 | 9.287 | 0.002 | 0.918 | 0.868 | 0.970 |
| **Others（Basic medical insurance for employees, Commercial medical insurance, Free medical treatment）** | -0.060 | 0.0319 | 3.591 | 0.058 | 0.941 | 0.884 | 1.002 |
| **Chronic diseases condition（control group =No chronic diseases）** |  |  |  |  |  |  |  |
| **Suffer from chronic diseases** | 0.071 | 0.0267 | 7.191 | 0.007 | 1.074 | 1.019 | 1.132 |
| **Monthly income (RMB )（control group =0-4500（0$-666$））** |  |  |  |  |  |  |  |
| **4501-9000（666.148$-1332$）** | -0.023 | 0.0223 | 1.091 | 0.296 | 0.977 | 0.935 | 1.021 |
| **＞9000（1332$）** | -0.054 | 0.0297 | 3.376 | 0.066 | 0.947 | 0.894 | 1.004 |
| **Extraversion（control group =High score group）** |  |  |  |  |  |  |  |
| **Low score group** | -0.023 | 0.0209 | 1.163 | 0.281 | 0.978 | 0.939 | 1.019 |
| **Agreeableness（control group =High score group）** |  |  |  |  |  |  |  |
| **Low score group** | -0.082 | 0.0224 | 13.440 | **＜0.001** | 0.921 | 0.881 | 0.962 |
| **Conscientiousness（control group =High score group）** |  |  |  |  |  |  |  |
| **Low score group** | -0.135 | 0.0229 | 34.617 | **＜0.001** | 0.874 | 0.835 | 0.914 |
| **Neuroticism（control group =High score group）** |  |  |  |  |  |  |  |
| **Low score group** | -0.021 | 0.0228 | 0.847 | 0.357 | 0.979 | 0.937 | 1.024 |
| **Openness（control group =High score group）** |  |  |  |  |  |  |  |
| **Low score group** | -0.044 | 0.0212 | 4.287 | 0.038 | 0.957 | 0.918 | 0.998 |
| **Health Literacy（control group =High score group）** |  |  |  |  |  |  |  |
| **Low score group** | -0.061 | 0.0233 | 6.937 | 0.008 | 0.941 | 0.899 | 0.984 |
| **EQ-VAS（control group =High score group）** |  |  |  |  |  |  |  |
| **Low score group** | -0.088 | 0.0222 | 15.648 | **＜0.001** | .916 | 0.877 | 0.957 |
